# Chloroplast genomic insights into adaptive evolution and rapid radiation in the genus *Passiflora* (Passifloraceae)

**DOI:** 10.1186/s12870-025-06210-9

**Published:** 2025-02-13

**Authors:** Luiz Augusto Cauz-Santos, Zirlane Portugal da Costa, Mariela Analía Sader, Cássio van den Berg, Maria Lucia Carneiro Vieira

**Affiliations:** 1 Department of Botany and Biodiversity Research, University of Vienna, Vienna, Austria; 2 Departmento de Genética, Escola Superior de Agricultura “Luiz de Queiroz”, Universidade de São Paulo, Piracicaba, SP Brazil; 3 Instituto Multidisciplinario de Biología Vegetal, Universidad Nacional de Córdoba, Córdoba, Argentina; 4 Departmento de Ciencias Biológicas, Universidade Estadual de Feira de Santana, Feira de Santana, BA Brazil

**Keywords:** Chloroplast genome, Genome rearrangements, Phylogenomics, Adaptive evolution, Rapid radiation, *Passiflora*

## Abstract

Chloroplasts are essential organelles in plants and eukaryotic algae, responsible for photosynthesis, fatty acid synthesis, amino acid production, and stress responses. The genus *Passiflora*, known for its species diversity and dynamic chloroplast (cp) genome evolution, serves as an excellent model for studying structural variations. This study investigates evolutionary relationships within *Passiflora* by sequencing 11 new chloroplast genomes, assessing selective pressures on cp genes, and comparing plastid and nuclear phylogenies. *Passiflora* cp genomes showed significant variations in size, gene content, and structure, ranging from 132,736 to 163,292 base pairs, especially in *Decaloba*. Structural rearrangements and species-specific repeat patterns were identified. Selective pressure tests revealed significant adaptive evolution in certain lineages, with several genes, including *clpP* and *petL*, under positive selection. Phylogenetic analyses confirmed the monophyly of subgenera *Astrophea*, *Passiflora*, and *Decaloba*, while *Deidamioides* appeared polyphyletic. Nuclear phylogenetic analysis based on 35S rDNA sequences supported the monophyly of *Astrophea* but showed inconsistencies within subgenus *Passiflora* compared to cp genome data. This study highlights the evolutionary complexity of *Passiflora* cp genomes, demonstrating significant structural variations and adaptive evolution. The findings underscore the effectiveness of plastid phylogenomics in resolving phylogenetic relationships and provide insights into adaptive mechanisms shaping cp genome diversity in angiosperms.

**Supplementary Information:**

The online version contains supplementary material available at 10.1186/s12870-025-06210-9.

## Introduction

Chloroplasts are essential organelles found in plants and some algae, playing critical roles in many crucial cellular processes, such as photosynthesis, fatty acid synthesis, amino acid production, and plant stress responses [41, 42, 83, 101]. This important organelle contains its own genetic material, distinct from the nuclear genome, which usually is organized as a circular DNA molecule, but in some cases is can be found as a linear structure [65]. The chloroplast genome (cp) is typically well conserved in angiosperms, ranging from 120 to 160 kilobases in size and encoding approximately 100 genes, including those genes encoding photosynthetic proteins and the ones encoding proteins used in the machinery for the cp gene expression [14, 82, 84].

Chloroplast comparative genomics can shed light on different evolutionary processes and functional adaptations, revealing, for example, the potential of cp genome structure variation in plant adaptation to abiotic stresses or different environment conditions [32, 77]. This comparative analysis can also be used to explore the evolutionary relationships among species, and reveal instances of horizontal gene transfer, gene duplication, and loss, offering a more comprehensive understanding of the dynamic nature of chloroplast genomes [66, 76], [12]. The evolution of next-generation sequencing (NGS) technologies is revolutionizing the field of comparative organelle genomics, allowing for a more detailed and extensive analysis of cp genomes across a wide range of the angiosperm phylogeny [79, 88, 104]. Recent studies are discovering a surprising level of diversity in cp genome structures, contradicting the earlier notion of their relative conservation. The significant variation in gene content, order, and in some cases presence of inverted repeats is suggesting that cp genomes are more dynamic and adaptable than previously understood, with evolutionary mechanisms such as rearrangements playing a crucial role in their diversity [26, 30]. Notable examples include the highly diverse chloroplast genomes found in groups such as Campanulaceae [29, 43], Geraniaceae [11, 89] and Papaveraceae [8]. Another angiosperm group with significant cp genome variability is *Passiflora*, known as passionflowers, which exhibits an extremely dynamic cp genome evolution, resulting in a high number of rearrangements, gene losses, and a particular case of loss of one inverted repeat [9, 10, 78]. All these events make this genus an excellent model for studying cp genome evolution.

*Passiflora* is the richest genus from the family Passifloraceae, comprising ca. 520 species with great diversity in size and shape of flowers. The geographic distribution of this group is particularly neotropical, being found mainly in the Americas; however, occurrences in Southeast Asia, Oceania and Australia have also been reported [87]. The first phylogenetic studies on this genus [61, 62, 97] resulted in poorly resolution of the *Deidamioides* clade, suggesting the paraphyletic position of this subgenus. Considering that plastid phylogenomics has been extensively used to solve the relationships in different plant groups [56, 59, 60, 94, 99], this analysis could also be useful to get new insights on the relationships wthin *Passiflora*. Clarfying, the current accepted taxonomic status of *Passiflora* is its subdivision into four subgenera, *Astrophea*, *Decaloba*, *Deidamioides* and *Passiflora*. However, with unresolved relationships within *Deidamioides* and the addition of *Tetraphatea*, it is critical to review these classifications.

In this study, we obtained 11 new plastomes for the genus *Passiflora* and conducted comparative genomics and phylogenetic inferences in order to investigate the evolutionary relationships between species within the genus. We also tested the robustness of the plastid phylogenetic analysis in comparison to nuclear rDNA phylogenies. Our aims were to answer: (i) What are the implications of structural variations in the cp genomes for understanding the *Passiflora* phylogenetic relationships? (ii) What do gene sequence comparisons in *Passiflora* cp genomes reveal about the impacts of gene variation and positive selection? (iii) What are the implications of positive selection in cp genes for the rapid radiation and diversification of the genus *Passiflora*? (iv) How the phylogeny from cp genomes compare to the one obtained from nuclear 35S rDNA in terms of resolving phylogenetic relationships within *Passiflora*?

## Material and methods

### Plant material and chloroplast (cp) DNA obtention

The present study includes 11 species of *Passiflora* comprising the subgenera: *Astrophea* (1). *Decaloba* (3), *Passiflora* (6) and *Tetrapathea* (1). Fresh leafs were collected from the Italian National Collection of *Passiflora*, Ripalta Cremasca, Italy (Supplementary Table 1), and prior to cpDNA extraction, the intact isolation of chloroplast organelles was performed based on liquid nitrogen-sucrose gradient method [85]. Briefly, in this procedure, approximately 10 g of fresh leaves were frozen in liquid nitrogen and macerated. The macerated leaves are then resuspended in 200 ml of isolation buffer, containing 50 mM Tris–HCl (pH 8.0), 0.35 M sucrose, 7 mM ethylenediaminetetraacetic acid (EDTA), 5 mM 2-mercaptoethanol, and 0.1% bovine serum albumin. This mixture was incubated in the dark for 10 min before being filtered through Miracloth, followed by centrifugation at 1,000 × g for 10 min to form a pellet. This pellet was washed and re-centrifuged under the same conditions. For further purification, the pellet was resuspended in isolation buffer and layered over a 20/45% sucrose gradient, then centrifuged at 2,000 × g for 30 min to isolate chloroplasts on the gradient interface. These chloroplasts were collected, diluted, and centrifuged at 3,000 × g to finally obtain a pellet of purified chloroplasts.

Subsequently, the resulted chloroplast pellet was lysed in 2% CTAB buffer, and the protocol was followed by two extractions using chloroform:isoamyl alcohol (24:1), an isopropanol precipitation, and finally washing the pellet in an ethanol solution (70%), that was then dried and resuspended in 40 μL of TE buffer.

### CpDNA sequencing and assembly

The cpDNA samples were used for long-read sequencing on the PacBio platform, and large-insert (10 kb) libraries were constructed using the Barcode method with 150 ng of pure high molecular weight DNA. The fragment sizes, quality, and DNA concentration of the libraries were checked using a Fragment Analyzer (Agilent Technologies) and Qubit 2.0 Fluorometer (Invitrogen). Sequencing was performed on two SMRT cells using P6 polymerase with C4 chemistry on a PacBio RS II platform at the NGI Platform (Uppsala, Sweden).

The raw data were demultiplexed and filtered based on quality, removing reads with a quality score below 0.75 and length shorter than 500 bp, then, the sequences were initially assembled and corrected using CANU assembler [44]. Chloroplast contigs were extracted in Geneious by aligning the assembled contigs to the complete *Passiflora* cp genomes available. The complete cp genomes were subsequently constructed in Geneious by connecting the contigs using the "de novo Assembly" function, and final assemblies were verified by mapping the raw reads to the final contig using the "Map to Reference" function in Geneious.

### Chloroplast genome annotation and comparative analysis

The new complete plastomes were annotated using the GeSeq (Organellar Genome Annotation) online program with default settings in order to identify protein-coding gene sequences (CDS), rRNAs and tRNAs based on cp reference sequences and BLAST homology searches [86]. The annotation was followed by manual corrections for start and stop codons, and intron positions in the GenomeView software,the OGDRAW software was used for constructing the circular and linear plastome maps [1, 28].

A multiple sequence alignment was performed to assess synteny and potential rearrangements among the cp genomes obtained. The progressive aligner from Mauve v.2.4.0 [15] was used to identify Locally Collinear Blocks (LCBs), which can reveal conserved and rearranged genomic regions across the species studied. Additionally, Mauve also put these blocks in order and orientation illustrating evolutionary and structural variations. We used CPJSdraw to illustrate the dynamic changes occurring in the inverted repeats (IRs) junction sites, in order to identify potential expansions and contractions of the IRs.

### Repeat identification

For the prediction of microsatellites, also known as Simple Sequence Repeats (SSRs), we used MISA-web [6]. The parameters for SSR search were defined as follows: Motifs ranging from one to six nucleotides in length, with minimum number of repeats set at 10 for mononucleotide, 5 for dinucleotide, and 4 for trinucleotide SSRs, and three repeats for tetra-, penta-, and hexanucleotide SSRs. Additionally, we used REPuter [50] to detect direct and palindromic repeated elements in the DNA sequences, with specific parameters set for a minimum repeat size of 30 base pairs and sequence identities of at least 90% (corresponding to a Hamming distance of 3).

### Plastid phylogenomic studies

Phylogenomic inferences were conducted based on 60 available plastomes from Passifloraceae (11 generated in this study, and 49 from species whose cp DNA sequences were obtained from NCBI’s Genbank). In addition, to obtain a rooted tree, the plastome of *Populus trichocarpa* (Salicaceae) was used as outgroup (Supplementary Table 1).

We used a set of 68 chloroplast protein-coding genes to perform the phylogenetic analysis: *atpA, atpB, atpE, atpF, atpH, atpI, ccsA, cemA, clpP, matK, ndhA, ndhB, ndhC, ndhD, ndhE, ndhF, ndhG, ndhH, ndhI, ndhJ, ndhK, petA, petB, petD, petG, petL, petN, psaA psaB, psaC, psaI, psaJ, psbA, psbB, psbC, psbD, psbE, psbF, psbH, psbI, psbJ, psbK, psbL, psbM, psbN, psbT, psbZ, rbcL, rpl2, rpl14, rpl16, rpl23, rpl33, rpl36, rpoB, rpoC1, rpoC2, rps2, rps3, rps4, rps8, rps11, rps12, rps14, rps15, rps19, ycf3, ycf4.*

The gene sequences were extracted from our data set (consisting in a total of 61 taxa), aligned individually at nucleotide level in MUSCLE, and then all the individual alignments were contatenated in a phylip matrix. The matrix was then analyzed in ModelFinder [37] to determine the best evolutionary model according to the Akaike Information Criterion (AIC). The Maximum Likelihood (ML) analysis was performed using RAxML version 8.2.4 [81]. The GTR + G + I model of nucleotide substitution was selected for the whole dataset, and a bootstrap analysis was performed with 1,000 replicates.

Additionally, a phylogenomic analysis using the complete plastomes of 24 species from subgenus *Passiflora* was performed (Supplementary Table 1). The sequences were aligned at nucleotide level in MAFFT version 7.221, using the FFT-NS-2 algorithm with default settings [39]. The substitution model for Bayesian inference (BI) was estimated in ModelFinder [37] and selected according to AIC. The Bayesian inference was conducted in MrBayes, with 10,000,000 generations, sampling one tree every 1,000 generations and discarding the first 25% of trees as burn-in. The analysis convergence was monitored using an average standard deviation of frequencies below 0.01, effective sample size (EES) above 200 for all parameters and potential scale reduction factor (PSRF) values close to 1.0. The phylogenetic trees from ML and BI inferences were visualized and edited using FigTree version 1.4.4 [70].

### Selective pressure analysis

We calculated the ratio of non-synonymous (*dN*) to synonymous (*dS*) substitutions (ω = *dN/dS*) for 68 cp protein-coding gene sequences. The coding sequences (CDS) from each gene was separately aligned using MUSCLE [18], with manual curation in Geneious. Subsequently, in order to identify genes potentially under positive selection, we calculated the *dN/dS* for each CDS alignment using the CODEML program in the PAML. While ω > 1 can suggest positive selection, it is essential that this ratio be statistically significantly greater than 1 before inferring adaptive evolutionary changes. In contrast, ω = 1 would indicates neutral selection, and ω < 1 points towards evidence of purifying selection [96]. Firstly, we used several models to estimate the selection pressure on genes: M0 (one ω), which assumes a single ω ratio for all sites; M1a (neutral), which allows for two categories of sites, one with ω = 0 and one with ω = 1; M2a (selection), which adds an additional category with ω > 1 to the M1a model, allowing for positive selection; M7 (beta), which assumes a beta distribution for ω across sites (0 < ω < 1); and M8 (beta & ω), which extends M7 by adding an extra category for ω > 1. The identification of positively selected sites was combined with the Naive Empirical Bayes (NEB) and the Bayesian Empirical Bayes (BEB) methods. To compare the models, we performed likelihood ratio tests (LRT) for M1a vs. M0, M2a vs. M1a, and M8 vs. M7, calculating the test statistic as twice the difference in log-likelihoods. The *p*-value was obtained from the chi-squared distribution of this statistic.

Additionally, to detect selection on specific lineages within the phylogenetic tree, particularly focusing on the clade of the genus *Passiflora*, we used branch models. We tested the one-ratio model, which assumes the same ω ratio for all branches, and the two-ratio model, which allows two different ω ratios: one for the foreground branches (specific to the *Passiflora* clade) and one for the background branches. We then performed the Likelihood Ratio Test (LRT) to determine whether the likelihood of the two-ratio model is significantly different from that of the one-ratio model by comparing two times the log likelihood difference. *P*-values were computed using a chi-square distribution with one degree of freedom [95].

### 35S rDNA sequence assembly and phylogenetic analysis

The complete 35S rDNA sequence was obtained by genome skimming from the sequencing data of 31 *Passiflora* species: 20 of them sequenced in a previous study using the Illumina next generation sequencing strategy [9], and 11 sequenced in this study using the PacBio sequencing approach (Supplementary Table 1). The de novo assembly of the 35S for each species was performed in NovoPlasty [16] for Illumina paired-end reads, and in Geneious for filtered reads generated with Pacbio. Annotation was conducted in Geneious and sequences were extracted to create two datasets for phylogenetic inferences based on 18S and 26S complete sequences.

Sequences were processed in a local pipeline, aligned individually in MUSCLE [18], and concatenated in an interleaved matrix. The best evolutionary model was estimated in ModelFinder [37] in accordance with AIC. The phylogenetic reconstruction was performed based on maximum likelihood (ML) analysis in RA × ML v.8.2.4 [81]. The GTR + G + I substitution model was selected to infer the phylogenetic relationships using non-parametric bootstrap analysis with 1000 repetitions.

Bayesian inference was performed using MrBayes v.3.2.5 [73] with GTR + G + I evolutionary model. Markov chain algorithm (MCMC) was conducted with 10,000,000 generations, sampling one tree every 1000 generations, with the first 25% of trees discarded as burn-in. The convergence of analysis was confirmed by an average standard deviation of frequencies below 0.01, effective sample size (ESS) above 200 for all parameters and potential scale reduction factor (PSRF) values close to 1.0. All trees were visualized and edited using the FigTree v.1.4.3 [70].

## Results

### Chloroplast genome structural features and gene content

Our analysis revealed significant variations in cp genome size, gene content, and structural organization. All *Passiflora* cp genomes obtained in the present study presented the typical quadripartite structure found in most angiosperms, consisting of a large single copy (LSC) region and a small single copy (SSC) region separated by two long inverted repeats (IRs) (Table 1, Supplementary Fig. 1). The genome sizes ranged from 132,736 bp in *P. adenopoda* (*Decaloba*) to 163,292 bp in *P. rusbyi* (*Astrophea*). The large single-copy (LSC) regions exhibited substantial size differences, from 47,752 bp in *P. intricata* (*Decaloba*) to 89,229 bp in *P. rusbyi* (*Astrophea*). However, while the SSC length was approximately 13 kb for all species analyzed, a large variation in the IR regions was observed. The IR regions, which are crucial for maintaining cp genome stability, showed variation of ca. 25 kb, spanning from 19,852 bp in *P. adenopoda* (*Decaloba*) to 55,553 bp in *P. intricata* (*Decaloba*). Regarding GC content, the cp genomes presented a relatively narrow range, from 36.2% in *P. tetrandra* (*Tetrapathea*) to 37.5% in *P. racemosa* (*Passiflora*). In total, the cp genome annotation resulted in 104 to 109 unique genes representing 70 to 75 protein-coding genes, 30 tRNA and 4 rRNA genes.

**Table 1:** Chloroplast genomic characteristics of 11 *Passiflora* species from different subgenera

Subgenus	Species	Cp genome (bp)	LSC (bp)	SSC (bp)	IR (bp)	GC content %	Unique genes	Protein coding genes	tRNAs	rRNAs
***Astrophea***	*Passiflora rusbyi*	163,288	89,229	12,839	30,610	36,6	107	73	30	4
***Decaloba***	*Passiflora adenopoda*	132,736	79,859	13,173	19,852	36,9	106	72	30	4
	*Passiflora intricata*	171,622	47,752	12,764	55,553	36,5	105	71	30	4
	*Passiflora xiikzozdz*	158,142	57,523	13,173	43,721	37,2	104	70	30	4
***Passiflora***	*Passiflora chaparensis*	150,928	85,062	13,492	26,187	37,1	106	72	30	4
	*Passiflora garckei*	146,299	86,007	13,240	23,526	37,1	106	72	30	4
	*Passiflora palenquensis*	149,524	85,083	13,495	25,473	36,9	106	72	30	4
	*Passiflora phoenicea*	148,089	85,806	13,495	24,394	36,9	106	72	30	4
	*Passiflora popenovii*	147,278	84,239	13,485	24,777	37,0	106	72	30	4
	*Passiflora racemosa*	159,892	86,642	13,940	29,625	37,5	106	72	30	4
***Tetrapathea***	*Passiflora tetrandra*	160,883	87,005	13,550	30,164	36,2	109	75	30	4

A comparison between the subgenera reveals notable infrasubgeneric variation in *Decaloba*, with an example of the IR size differences between *P. adenopoda* and *P. intricata*, suggesting significant genomic restructuring in this subgenus. Meanwhile, the subgenus *Passiflora* shows consistent gene content across its species, and a variability of ca. 6 kb for the IR sizes in the species analyzed.

### Comparative genomics

**Fig. 1:**
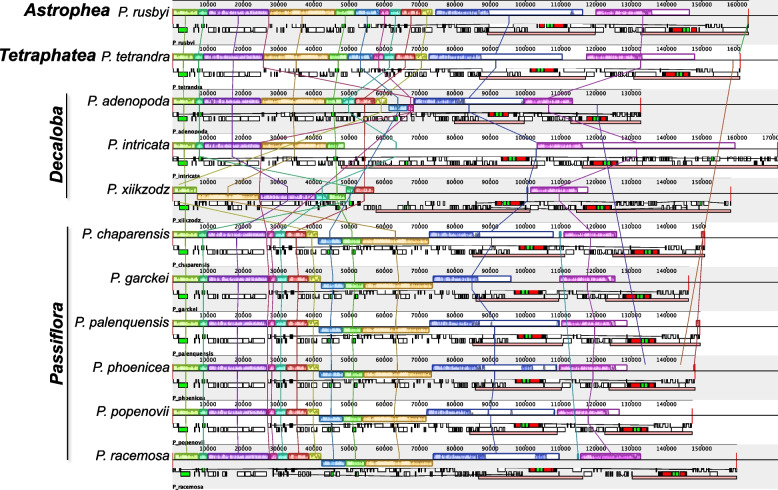
Comparative analysis of cp genomes across different subgenera in *Passiflora*. The alignments were obtained in Mauve, and colored bars represent syntenic blocks across the genomes, while connecting lines show the correspondence between these blocks. Below the synteny blocks, gene annotations for each chloroplast genome are displayed, with rRNA genes highlighted in red. Additionally, the inverted repeat (IR) regions are marked as light red bars in the annotation section

The comparative analysis of the cp genomes across different *Passiflora* species revealed distinct patterns of genomic organization, with 10 synteny blocks (LCBs) or conserved regions among the cp genomes aligned. In general, rearrangements characterized by inversions, IR expansions and contractions were detected (Fig. 1).

We found a similar structure in the cp genomes of *P. rusbyi* and *P. tetrandra*, representing the subgenera *Astrophea* and *Tertraphatea*. No major rearrangements could be observed between these two species, which only differ by an increase of ca. 2 kb in the LSC region in *P. rusbyi.* In contrast, subgenus *Decaloba* demonstrates extensive genomic rearrangements, for instance, *P. intricata* exhibits notable expansions and contractions in several LCBs, representing mainly an expansion of ca. 25 kb in the inverted repeat regions compared with *Astrophea* and *Tertraphatea*. Additionally, *Decaloba* exhibited the highest infrasubgeneric variation, with the IRs varying in ca. 35 kb among the species. Apart from that, when comparing *P. intricata* with the other species within the same subgenus, *P. adenopoda and P. xiikzodz*, we found inversions and translocations as variations in number and structure of LCBs, suggesting a dynamic organellar genome evolution in this subgenus. Within the species of the subgenus *Passiflora*, the cp genomes maintain a high degree of synteny, and only expansions of the IRs were observed as rearrangements. The largest IR expansion, observed in *P. racemosa*, is approximately 6 kb larger than the smallest IR found in *P. garckei*. However, in comparison with other subgenera, an inversion of approximately 30 kb in the LSC region is observed among the species of subgenus *Passiflora*.

When comparing the junctions of the quadripartite structure across the cp genomes in different *Passiflora* subgenera, distinct patterns of gene arrangement were found (Supplementary Fig. 2). In *Astrophea*, represented by *P. rusbyi*, the gene boundaries at the junctions between the inverted repeats (IRs) and the small single-copy (SSC) regions show an IR expansion, incorporating a copy of the *ndhH* gene. This configuration is different from most other species, where the *ycf1* gene typically is in the IRs boundaries. *Decaloba* is the most variable subgenus in terms of gene arrangements at the junctions, a variation largely resulting from observed rearrangements, including IR expansion and contraction. *P. adenopoda* exhibits a contraction of the IR, leading to *rps3* gene spanning from the IRb into the LSC. On the other hand, *P. intricata* shows a large IR expansion, incorporating several genes found in other species in the LSC region, extending up to the *ndhC* and *ndhK* genes. Additionally, *P. xiikzodz* displays distinct gene placement with *psaI* located at one of the IR borders, and *ycf2* spanning between the IR and SSC junction. In contrast to the variability observed in *Decaloba*, species within the subgenus *Passiflora* generally exhibit a conserved gene order at the junctions. However, a slight difference is observed, particularly with the *rps15* gene, which in some species of this subgenus spans from the IR to the SSC region.

### Repeat distribution

**Fig. 2:**
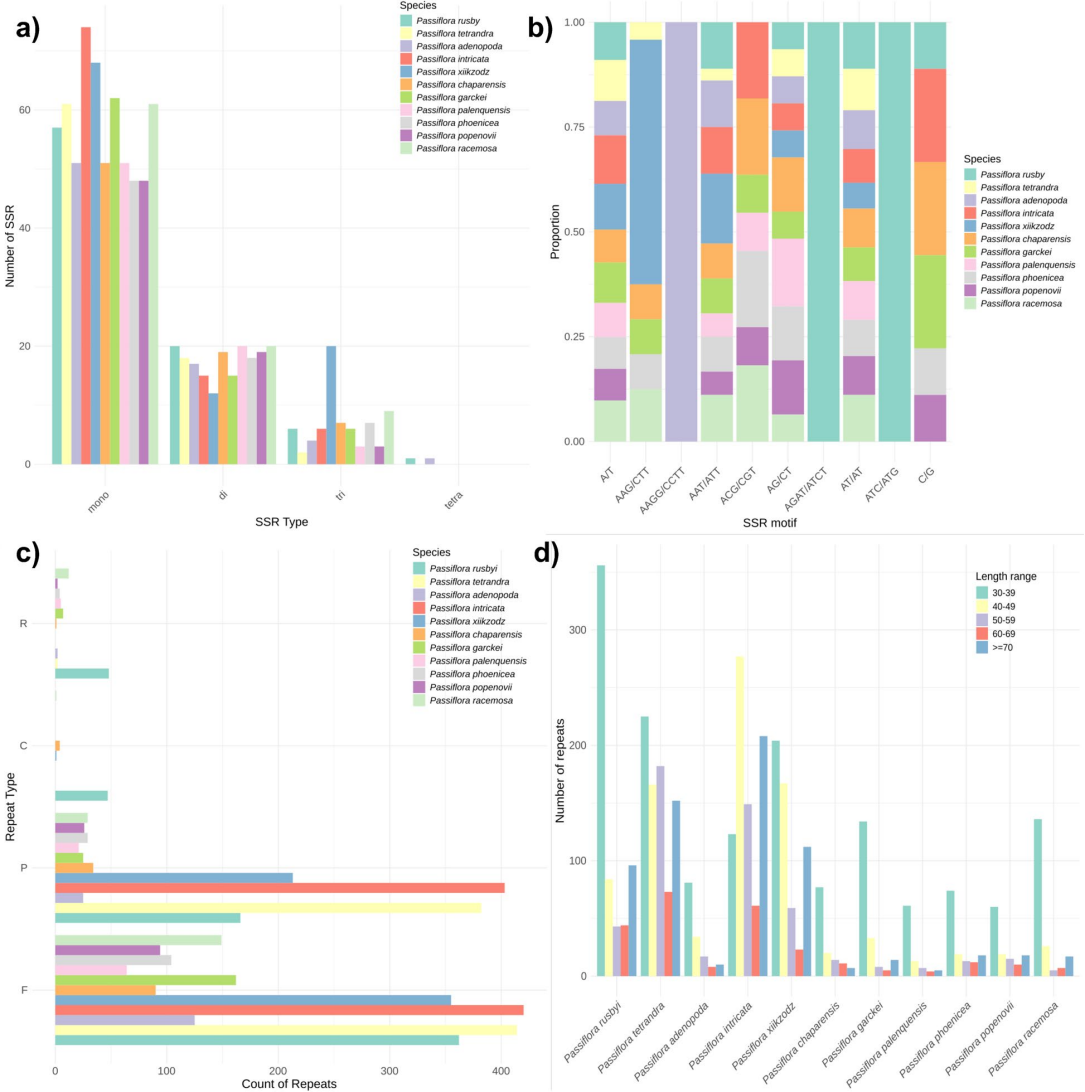
Distribution and characteristics of repeats in *Passiflora* cp genomes. a) Distribution of SSR types (mono, di, tri, tetra, penta, hexa) across different *Passiflora* species; b) Proportional representation of SSR motifs within each species; c) Number of repeats categorized by repeat types (F: Forward, P: Palindrome, C: Complement, R: Reverse); d) Number of repeats within specified length ranges (30–39, 40–49, 50–59, 60–69, ≥ 70) for each *Passiflora* species

The distribution and characteristics of repeats in *Passiflora* cp genomes reveal distinct patterns across different species and subgenera (Fig. 2). The identification of simple sequence repeat (SSR) types revealed that mono and dinucleotide repeats are the most prevalent types across all species (Fig. 2a), with the highest numbers of mononucleotide repeats observed in *P. intricata* and *P. xiikzodz*, both species from subgenus *Decaloba*. The number of trinucleotide repeats varied, with the highest number in *P. intricata* and the lowest in *P. tetrandra*. Furthermore, the occurrence of tetranucleotide repeats is significantly lower, only one repeat was identified in both *P. rusbyi* and *P. adenopoda*, and no penta- or hexanucleotide repeats were found in any of the species. Regarding the proportional representation of SSR motifs (Fig. 2b), the A/T motif is the predominant in most species showing similar proportions. Other motifs like C/G, AG/CT, AAG/CTT, and ACG/CGT show varying proportions, indicating species-specific preferences for certain SSR motifs.

We also identified long sequence repeats (> 30 bp) from the types: Forward (F), Palindrome (P), Complementary (C), and Reverse (R) (Fig. 2c). The most common repeat type across all species was Forward, with *P. tetrandra* (subgenus *Tetraphatea*) and *P. intricata* (subgenus *Decaloba*) showing the highest counts, 414 and 420 respectively. Palindromic repeats were also highly represented, with these same species having the highest counts, while species from subgenus *Passiflora* presented similar count values, ca. 30. The complementary and reverse are the least commonly found repeats, apart from the 48 reverse repeats found in *P. rusbyi* (subgenus *Astrophea*). Repeats within the range of 30–39 bp are the most prevalent across most species (Fig. 2d), with *P. rusbyi* and *P. tetrandra* showing particularly high frequencies. However, in *P intricata*, the 40–49 bp range is the most prevalent. Longer repeats (> 70 base pairs) are relatively uncommon in subgenus *Passiflora* but were found in high frequency in two species of the subgenus Decaloba and in both species from the subgenera *Astrophea* and *Tetrataphea*.

### Plastid phylogenomics

**Fig. 3:**
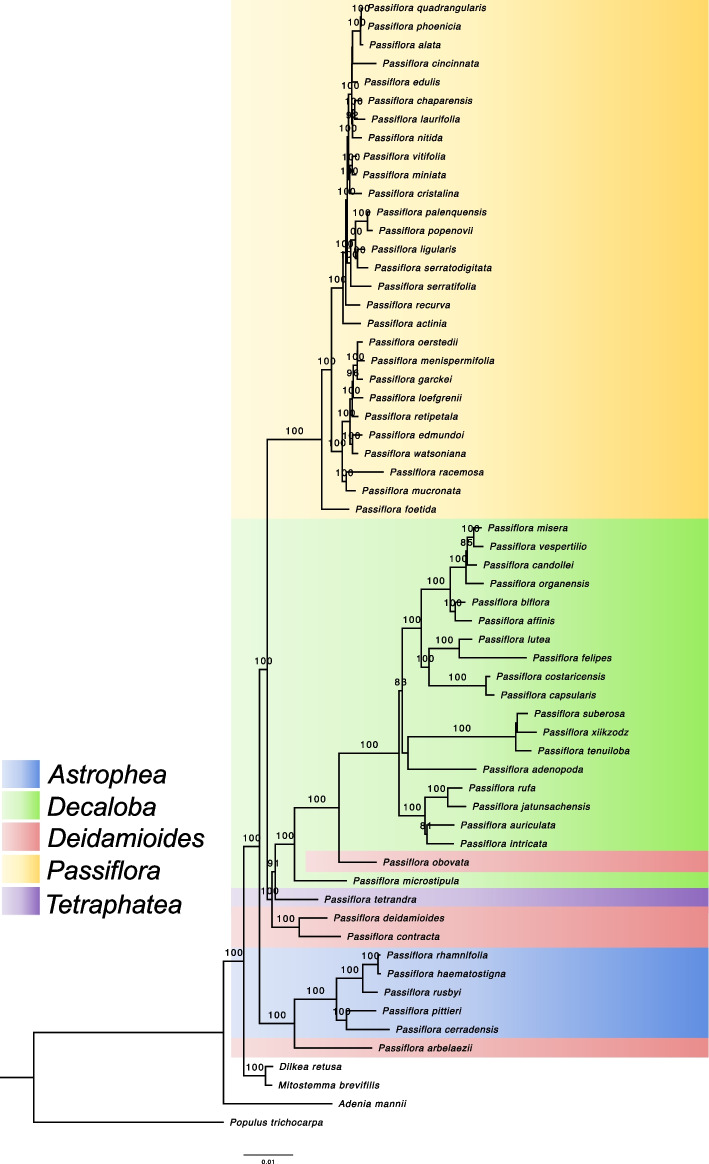
RAxML phylogenetic reconstruction of *Passiflora* evolutionary history based on 68 chloroplast protein-coding genes under the GTR + G + I substitution model. Node supports are indicated by bootstrap values (BS)

The final alignment matrix of the plastid protein-coding genes was 59,350 characters long, and the ML analysis resulted in a tree with high support (BS = 100) for most of the nodes (Fig. 3). We used *Populus trichocarpa* (Salicaceae) to root the tree, and the base of the tree we found the other Passifloraceae species, *Adenia manii*, *Dilkea retusa* and *Mitostemma brevifilis* with high support (BS = 100).

Regarding the formerly described *Passiflora* subgenera, our results revealed the monophyly of *Astrophea*, *Passiflora* and *Decaloba.* In *Astrophea*, a clade consisting of *P. haematostigma* and *P. rhamnifolia* species (section *Pseudoastrophea*) was found, grouping as sister to *P. rusbyi* (BS = 100) from the section *Botryastrophea*. On the other hand, the species from section *Capreolata* (*P. cerradensis* and *P. pittieri*) grouped together (BS = 100). Our results show the polyphyletic positioning of species from subgenus *Deidamioides*, while *P. arbelaezii* (section *Tryphostemmatoides*) formed a clade (BS = 100) which is sister to *Astrophea*, *P. obovata*, in subgenus *Deidamioides*, was embedded in subgenus *Decaloba*. In addition, another group consisted of *Deidamioides* species was observed: *P. contracta* from section *Tetrastylis* and *P. deidamioides* from section *Deidamioides* (BS = 100) appeared as monophyletic and sister to *Decaloba.*

The species from subgenus *Decaloba* formed a monophyletic group; however, *P. microstipula* grouped with *P. obovata* (*Deidamioides*). A clade with high support (BS = 100) was observed consisting of species of supersection *Auriculata* (*P. jatunsachensis*, *P. rufa*, *P. intricata* and *P. auriculata*). The species of supersection *Cieca* (*P. xiikzodz*, *P. suberosa* and *P tenuiloba*) grouped together and sister to supersection *Bryonoides*. In our study, section *Decaloba* appears as paraphyletic, with the group of *P. filipes* and *P. lutea* as sister to the species of section *Xeragona* (*P. capsularis* and *P costaricensis*). In addition, *P. tetrandra,* the type species of subgenus *Tetrapathea*, grouped as sister to subgenus *Decaloba*.

Our analysis included 28 species from subgenus *Passiflora*, confirming the monophyly of this clade. However, when comparing the distinct supersections within it, paraphyly was observed. In the tree, two major clades with high support (BS = 100) were found in subgenus *Pasiflora*, one consisted of species from supersection *Stipulata*, and the other comprising species from different supersections (*Coccinea*, *Distephana*, *Laurifolia*, and *Passiflora*). Although most species from *Stipulata* grouped together as a single cluster, this supersection is paraphyletic with *P. actinia* grouping closer to the species from supersection *Laurifolia* and *Passiflora*.

Regarding the relationships within the supersection *Stipulata*, the placement of species into some sections appeared paraphyletic, *P. loefgrenii* is from section *Kermesinae*, the same of *P. edmundoi* and *P. watsoniana*; however, in our findings, it was embedded in section *Granadillastrum*, a clade formed by *P garckei*, *P. menispermifolia*, *P. oerstedii* and *P. retipetala*. The supersection *Passiflora* also appeared as polyphyletic: *P. cincinnata*, *P. edulis, P. recurva* and *P. serratifolia* that belong to the same series in this supersection grouped each one with different sub-clades with species from supersection *Laurifolia*. In addition*, P. vitifolia*, from supersection *Coccinea,* grouped with *P. miniata* and *P. cristalina* from the supersection *Distephana* with high support (BS = 100)*.*

To compare the results with the clustering into supersections of subgenus *Passiflora*, a phylogenomic analysis was performed based on whole cp genome sequences (Supplementary Fig. 3). The tree topology was congruent with the one based on the cp genes, with the supersections *Passiflora* and *Laurifolia* as paraphyletic.

### Estimation of *dN/dS* ratios and positive selection

**Fig. 4:**
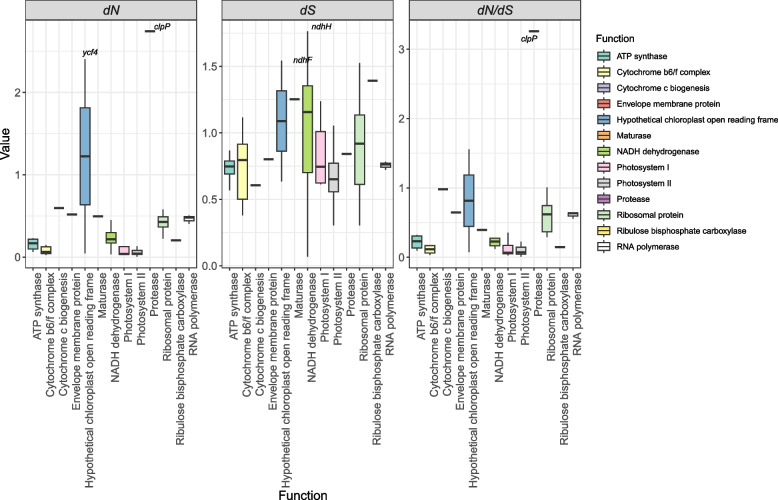
Distribution of *dN*, *dS* and *dN*/*dS* (nonsynonymous/synonymous substitution rate) for 68 cp protein-coding genes obtained from codeml under model M0. The genes were grouped per chloroplast function categories. Each box represents the interquartile range (IQR), showing the middle 50% of the data, with the horizontal line within each box corresponding to the median values for the corresponding category. Outliers are labeled with gene names

We estimated the ratio of non-synonymous (*dN*) to synonymous (*dS*) substitutions was performed for 68 cp protein-coding genes from 61 species (57 from genus *Passiflora*). Various site models (M0, M1a, M2a, M7, and M8) were employed to compare model fit using likelihood ratio tests (LRTs). Of these, only comparisons of M1a vs. M2a and M7 vs. M8 were used to specifically test for the presence of positive selection. Considering the *dN/dS* ratios of Model 0, most of the genes were identified under purifying selection (*dN/dS* ratio < 1), indicating selective pressure to maintain their functions (Fig. 4). However, when comparing the models, the results showed several genes with sites evolving under positive selection, as evidenced by the log-likelihood values and the likelihood ratio test (LRT) results (Supplementary Table 2). For instance, the *atpA* shows a significant result with log-likelihood values of -5370.334007 for M2a and -5416,734,687 for M1a, which resulted in a likelihood ratio test statistic of 92.801360, indicating sites under strong evidence of positive selection (Supplementary Table 2). Other genes such as *atpB*, *atpE*, *atpF*, and *ccsA* also exhibit sites under significant positive selection with *p*-values well below the threshold of 0.05. Functionally, the genes under positive selection belong to categories that are crucial for cellular and metabolic processes. The *atp* genes (*atpA*, *atpB*, *atpE*, *atpF* and *atpH*) are involved in ATP synthesis, which is essential for cellular energy production. The *ndh* genes (*ndhA*, *ndhC*, *ndhF*, *ndhI*, *ndhK*) are part of the NADH dehydrogenase complex, which plays a key role in the electron transport chain and cellular respiration. Additionally, genes like *rpoB*, *rpoC1*, and *rpoC2*, which encode components of the RNA polymerase complex, showed very high likelihood ratio test statistics and significant *p*-values (for example, *rpoB* with a test statistic of 232.414876), indicating strong positive selection in these fundamental transcriptional machinery genes.

The likelihood ratio tests (LRT) for positive selection using the branch-site model with subgenus *Passiflora* as the foreground branch reveal strong evidence of positive selection for several genes (Table 2). Specifically, the genes *atpA*, *atpB*, *ccsA*, *clpP*, *matK*, *petA*, *petD*, *petL*, *psaA*, *psbA*, *psbF*, *rbcL*, *rpl16*, *rpoC1*, *rpoC2*, *rps15*, and *rps3* show significant LRT statistics and *p*-values (*p* < 0.05), indicating that they have undergone adaptive evolution. For instance, the *rbcL* gene shows a highly significant *p*-value of 1.45e-9. Similarly, *rpoC2* with a *p*-value of 2.57E-10 and *atpB* with a *p*-value of 6.27e-5 also indicate a robust evidence for positive selection.

**Table 2:** Likelihood ratio test (LRT) results for positive selection in cp genes using the branch-site model from codeml and assuming the foreground branch as subgenus *Passiflora*. The null model assumes no positive selection, whereas the alternative model allows for positive selection on the foreground branch. A significant LRT statistic and *p*-value (< 0.05) indicate evidence for positive selection

Gene	Null Model (lnL)	Alternative Model (lnL)	LRT	*P* value	Positive selection
***atpA***	**-5416.731061**	**-5412.139146**	**9.183.830.000.000.070**	**0e0**	**True**
***atpB***	**-4653.663776**	**-4632.246694**	**4.283.416.399.999.890**	**0e0**	**True**
***atpE***	**-1191.894703**	**-1191.132408**	**1.524.589.999.999.850**	**0e0**	**True**
***atpF***	**-2936.756742**	**-2936.616616**	**2.802.520.000.003.270**	**6.62e-63**	**True**
*atpH*	-557.53033	-557.53033	0.0	1.00e0	False
*atpI*	-1875.054947	-1875.054947	0.0	1.00e0	False
***ccsA***	**-3482.990348**	**-3465.913442**	**3.415.381.200.000.080**	**0e0**	**True**
*cemA*	-3022.996837	-3022.996837	0.0	1.00e0	False
***clpP***	**-1268.888758**	**-1248.732428**	**4.031.265.999.999.960**	**0e0**	**True**
***matK***	**-7323.240373**	**-7316.877636**	**12.725.473.999.999.400**	**0e0**	**True**
***ndhA***	**-3537.27328**	**-3536.501507**	**1.543.545.999.999.150**	**0e0**	**True**
*ndhB*	-2501.650325	-2501.650326	-0.0019999993965029716	1.00e0	False
*ndhC*	-1081.116279	-1081.116279	0.0	1.00e0	False
***ndhD***	**-5493.044036**	**-5492.610016**	**8.680.400.000.009.680**	**8.70e-191**	**True**
*ndhE*	-1006.688506	-1006.688506	0.0	1.00e0	False
***ndhF***	**-11,525.529656**	**-11,525.491883**	**755.460.000.000.894**	**3.57e-18**	**True**
***ndhG***	**-1975.749812**	**-1975.266312**	**967.0**	**2.68e-212**	**True**
*ndhH*	-4165.476882	-4165.476882	0.0	1.00e0	False
***ndhI***	**-1745.53302**	**-1745.009447**	**10.471.460.000.001.800**	**1.02e-229**	**True**
*ndhJ*	-1238.696502	-1238.696502	0.0	1.00e0	False
*ndhK*	-2009.235367	-2009.235361	0.012000000104308128	9.13e-1	False
***petA***	**-3048.800084**	**-3046.458667**	**4.682.833.999.999.790**	**0e0**	**True**
*petB*	-1793.004653	-1793.004653	0.0	1.00e0	False
***petD***	**-1297.723744**	**-1295.681768**	**40.839.520.000.000.400**	**0e0**	**True**
*petG*	-217.777657	-217.777657	0.0	1.00e0	False
***petL***	**-330.530003**	**-319.888169**	**21.283.668.000.000.000**	**0e0**	**True**
*petN*	-185.581145	-185.581145	0.0	1.00e0	False
***psaA***	**-5526.801735**	**-5523.994622**	**5.614.225.999.999.790**	**0e0**	**True**
*psaB*	-5221.967328	-5221.967338	-0.020000001415610313	1.00e0	False
***psaC***	**-651.040484**	**-649.461902**	**3.157.164.000.000.100**	**0e0**	**True**
*psaI*	-426.171134	-426.171134	0.0	1.00e0	False
***psaJ***	**-405.111325**	**-403.265708**	**3.691.234.000.000.050**	**0e0**	**True**
***psbA***	**-2879.213041**	**-2873.140644**	**12.144.794.000.000.600**	**0e0**	**True**
*psbB*	-3969.137739	-3969.137739	0.0	1.00e0	False
*psbC*	-3405.428198	-3405.428198	0.0	1.00e0	False
*psbD*	-2203.354638	-2203.354637	0.0019999993965029716	9.64e-1	False
*psbE*	-449.236923	-449.236923	0.0	1.00e0	False
***psbF***	**-217.278231**	**-214.649417**	**5.257.628.000.000.020**	**0e0**	**True**
***psbH***	**-623.084017**	**-622.001225**	**21.655.840.000.000.300**	**0e0**	**True**
***psbI***	**-198.445739**	**-198.094300**	**7.028.780.000.000.260**	**7.08e-155**	**True**
***psbJ***	**-249.380412**	**-248.211244**	**23.383.360.000.000.100**	**0e0**	**True**
*psbK*	-628.334431	-6288.78510	-1.088.158.000.000.050	1.00e0	False
*psbL*	-220.155601	-220.155601	0.0	1.00e0	False
*psbM*	-209.740165	-209.740165	0.0	1.00e0	False
*psbN*	-291.213313	-291.213313	0.0	1.00e0	False
*psbT*	-197.355030	-197.355.030	0.0	1.00e0	False
*psbZ*	-390.653136	-390.653136	0.0	1.00e0	False
***rbcL***	**-5756.967145**	**-5725.125326**	**63.683.637.999.998.400**	**0e0**	**True**
*rpl14*	-1743.031898	-1743.031898	0.0	1.00e0	False
*rpl16*	-1444.679184	-1444.679184	0.0	1.00e0	False
*rpl2*	-2389.389656	-2389.389656	0.0	1.00e0	False
*rpl23*	-1020.163902	-1020.163902	0.0	1.00e0	False
***rpl33***	**-762.190101**	**-762.101070**	**17.806.200.000.015.000**	**1.28e-40**	**True**
*rpl36*	-474.175797	-474.175797	0.0	1.00e0	False
***rpoB***	**-13,348.809868**	**-13,348.327628**	**964.480.000.000.447**	**9.44e-212**	**True**
***rpoC1***	**-9121.134828**	**-9109.852291**	**22.565.074.000.000.900**	**0e0**	**True**
***rpoC2***	**-17,383.464617**	**-17,349.835137**	**672.589.600.000.009**	**0e0**	**True**
*rps11*	-2454.706719	-2454.706719	0.0	1.00e0	False
***rps12***	**-784.166964**	**-746.559065**	**7.521.579.800.000.010**	**0e0**	**True**
*rps14*	-1115.535563	-1115.535563	0.0	1.00e0	False
***rps15***	**-1533.141721**	**-1530.759860**	**4.763.721.999.999.600**	**0e0**	**True**
***rps19***	**-959.329801**	**-958.189129**	**2.281.344.000.000.040**	**0e0**	**True**
*rps2*	-3380.312648	-3380.312648	0.0	1.00e0	False
***rps3***	**-3196.291978**	**-3193.633343**	**5.317.270.000.000.480**	**0e0**	**True**
*rps4*	-2550.391045	-2550.391045	0.0	1.00e0	False
*rps8*	-1734.476247	-1734.476247	0.0	1.00e0	False
*ycf3*	-1172.304022	-1172.304022	0.0	1.00e0	False
*ycf4*	-2492.693465	-2492.693467	-0.0040000006556510925	1.00e0	False

### Nuclear phylogeny based on complete 18S/26S (35S rDNA cistron) gene sequences

**Fig. 5:**
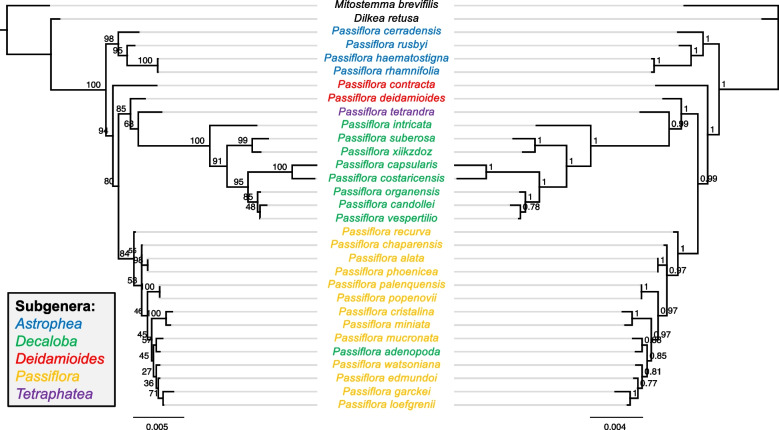
Maximum Likelihood and Bayesian phylogenetic inference of *Passiflora* genus based on the nuclear 18S/26S gene sequences

Through genome skimming, the sequencing of *Passiflora* cpDNAs enabled the assembly of the complete 35S rDNA cistron, which was used to perform a phylogenetic analysis based on the complete 18S and 26S gene sequences. The phylogenetic tree using both BI and ML methods resulted in similar topologies (Fig. 5).

The results strongly support the monophyly of subgenus *Astrophea* (BS = 98, PP = 1). However, despite the high values that support the grouping of subgenera *Passiflora* (BS = 84, PP = 1) and *Decaloba* (BS = 100, PP = 1), the monophyly of these subgenera was not observed, since *P. adenopoda* (*Decaloba*) was embedded within subgenus *Passiflora*. Additionally, the subgenus *Deidamioides* appears paraphyletic, with *P. deidamioides* emerging as sister to *P. tetrandra* (*Tetraphathea*). While the phylogeny provides high bootstrap support for higher classification levels such as subgenera, it reveals low bootstrap support for numerous nodes within subgenus *Passiflora*, indicating unresolved relationships. The tree also shows a polytomy and very short branches, which contributed to the lack of resolution for species such as *P. chaparensis*, *P. alata*, and *P. phoenicea*. Furthermore, incongruences between different trees (Figs. 3 and 5), generated by two distinct datasets, were primarily observed in the clustering within subgenus *Passiflora*. Unlike the nuclear phylogeny, in the cp genome tree, *P. adenopoda* was placed into the *Decaloba* clade, highlighting the differences in classification derived from the different genomes.

## Discussion

### Structural rearrangements in *Passiflora* chloroplast genomes

In the past, chloroplast genomes of angiosperms were known to be highly conservative in structure and gene order, but studies on comparative genomics have been revealing that this is not always the case [8, 89, 93]. *Passiflora*, a genus within the Passifloraceae family, is an example of the diversity and complexity of cp genome structure and evolution, with significant structural rearrangements, including inversions, duplications, IR expansions/contractions, and even losses of certain gene regions [9, 10, 69, 78].

Our results confirm the highly dynamic evolution of cp genomes in this genus, as we identified gene losses and large-scale inversions, such as inversions and IR expansions/contractions, in the newly assembled cp genomes. For instance, the large inverted repeat regions (IRs), which are usually quite stable in most angiosperms, show considerable variation in *Passiflora* species. Our analysis resulted in an IR size difference of almost 35 kb in the subgenus *Decaloba*, leading to large scale cp gene rearrangements in the species of this subgenus. It is hypothesized that inverted repeat regions are crucial for maintaining cp genome stability [55, 67], and a recent study by Krämer et al. [45] demonstrates that the removal of these regions can lead to a reduced number of plastid ribosomes and an increased total number of plastid genomes, highlighting the functional importance of these regions. In general, the IRs of *Passiflora* exhibit a huge variation in their evolutionary history, which in some cases has led to higher substitution rates for genes incorporated into IR expansions [78], and in the most extreme case has resulted in the loss of one inverted repeat copy [9]. One of the potential sources of the cp genome rearrangements is the repeat sequences, which we found to be abundant in the cp genome of some *Passiflora* species. Repeats can facilitate recombination, a process that could result in rearrangements, such as inversions, duplications and deletions [55, 63, 72, 93].

The study of cp genome structure could significantly help resolving the evolutionary relationships among plant species. The cp genome rearrangements have been identified as potentially valuable for plant taxonomic studies [17, 34, 71]. Particularly, the cp gene order has proven to be a good marker for phylogenetic studies in Campanulaceae [13]. Additionally, in legumes, the loss of an inverted repeat (IR) region has enabled the classification of an extensive number of papilionoid genera into what is described as the Inverted Repeat-Lacking Clade (IRLC) [51, 58, 91]. Similarly, the rearrangements we identified could be a rich source of markers for circumscribing species and tracing the evolutionary histories in *Passiflora*.

### Passiflora plastid phylogenomics

Plastid phylogenomics has become a valuable tool in plant taxonomy, using both a set of genes [33, 94] or the whole cp genome sequences [35, 100]. The increasing number of available cpDNA sequences and the application of phylogenomics have significantly enhanced the accuracy of phylogenies, resulting for example, in higher tree resolution at low taxonomic levels [53, 68, 92, 102].

*Passiflora* has a complex taxonomic history, with Killip [40] first proposing the division into 22 subgenera based on morphological traits. This number was later drastically reduced to four by Ulmer & MacDougal [87], who, considering both morphological and ecological information, suggested the existence of the subgenera *Astrophea*, *Decaloba*, *Deidamioides*, and *Passiflora*. This reduction highlights the significant morphological diversity within *Passiflora* species, leading to considerable distinctions between the subgenera. For example, *Decaloba* (ca. 230 species) are herbaceous vines with small fruits and flowers, whilst *Passiflora* (ca. 250 species) are lianas or herbaceous vines with large, colorful flowers and long tubes. *Deidamioides* (14 species) are characterized by tiny stipules, petiolar glands, small bracts, and a plicate operculum [87]. *Astrophea* (ca. 60 species) consists of woody lianas, shrubs, or small trees lacking tendrils or presenting short spines. This morphological diversity is mirrored in their pollination strategies. For instance, species in the supersection *Tacsonia* are adapted for hummingbird pollination [2], while others, such as those of the series *Tetrastylis* (*Deidamioides*), are pollinated by small and large insects and bats [7, 22]. Despite their predominantly neotropical distribution, 22 species from supersection *Disemma* (*Decaloba*) are found in Southeast Asia, Australia and New Zealand [47].

To address these taxonomic complexities, Feuillet and MacDougal [21] proposed subdivisions within the four subgenera into supersections. Further supporting this effort, the first molecular phylogeny based on nuclear and cpDNA markers revealed three well-supported clades: *Astrophea*, *Decaloba*, and *Passiflora*, but did not resolve the relationships in *Deidamioides* [61]. Subsequent studies also recovered *Deidamioides* as polyphyletic, proposing eight subgenera [97]. Our plastid phylogenomic analysis also supports these findings, revealing high support (BS = 100) for most nodes and confirming the monophyly of *Astrophea*, *Passiflora*, and *Decaloba*. Further adding to the complexity, Krosnick et al. [46] proposed *Tetrapathea* as a new subgenus, represented in this study by *P. tetrandra*. In our analysis, *Tetrapathea* was placed as a sister to *Decaloba*, corroborating previous phylogenetic findings [48].

Our trees also indicate the polyphyletic nature of subgenus *Deidamioides*, with *P. arbelaezii* forming a clade sister to *Astrophea*, *P. obovata* embedded within subgenus *Decaloba*, and *P. contracta* + *P. deidamioides* clustering as sisters to *Decaloba*. These findings suggest that the current taxonomic classification of *Deidamioides* does not accurately reflect its evolutionary history. Given these complexities, our results highlight the need for a comprehensive revision of subgenus *Deidamioides*. A more detailed phylogenetic study including additional nuclear markers and broader taxon sampling could provide deeper insights into the evolutionary relationships within this group to allow a revised circumscription. Within subgenus *Passiflora*, our analysis including 28 complete cp genomes resulted in paraphyly observed within distinct supersections, as in supersection *Stipulata* and *Passiflora*. This pattern within subgenus *Passiflora* may be due to recent divergence and rapid radiations in this subgenus, which would result in incomplete lineage sorting impacting the resolution of relationships in lower taxonomic levels.

### Adaptive evolution in *Passiflora* cp genomes

After unraveling the evolutionary dynamics of *Passiflora* cp genomes, our phylogenetic study supports the occurrence of a rapid radiation in this genus, an event where species diversify quickly from a single ancestor into an extensive range of species. Importantly, rapid radiation is driven by ecological opportunities, which often leads to selective pressure that shape genomic adaptations. By examining the ratio of non-synonymous (*dN*) to synonymous (*dS*) divergence (*dN/dS*), we found that the majority of genes exhibited overall *dN/dS* ratios < 1, indicating that purifying selection predominates, a pattern which does not exclude the presence of positively selected sites within these genes. Notably, certain genes, including *clpP*, presented overall *dN/dS* ratios significantly greater than 1, providing strong evidence for positive selection acting on at least a subset of their codons.

The high *dN/dS* ratios for *clpP* gene found in our results was also observed previously for other angiosperm groups, such as *Oenothera*, *Silene* [19] and *Acacia* [90]. In *Passiflora,* the evolution of *clpP* gene is also marked by two independent events of intron loss in subgenera *Decaloba* and *Passiflora* [9]. The chloroplasts Clp proteases are crucial for protein degradation and plant development. The disruption of *clpP* gene, encoding the ClpP1 subunit, caused severe developmental defects in mutant tobacco plants, including shoot system loss [49]. Shikanai et al. [80] showed that *clpP* gene is vital for chloroplast biogenesis, affecting early developmental stages in leaf primordia and the maturation of grana, by degrading denatured proteins and rapidly turning over regulatory factors. Additionally, evidence from a study in *Arabidopsis thaliana* reveals the involvement of ClpP in the latter stages of both high light and cold shifts, suggesting a role in acclimation to changing growth conditions [103].

Most surprisingly, the branch-site model test for positive selection revealed several genes under adaptive evolution in the subgenus *Passiflora*. The genes identified under positive selection include those involved in photosynthesis (*psaA*, *psbA*, *rbcL*), transcription and translation (*rpoC1*, *rpoC2*, *rps15*), and metabolic processes (*clpP*, *ccsA*). Particularly, subgenus *Passiflora*, which includes more than 230 species, has shown signs of rapid radiation in previous studies, with a diversification rate of 12 species per million year [62, 74]. Interestingly, the *petL* gene, which encodes a small subunit of the cytochrome b6f complex, also showed evidence of positive selection. A recent study has demonstrated that PetL is crucial for photosynthetic cold acclimation, highlighting its role in enabling plants to adapt to cold environments [27]. This is particularly relevant as some species of the subgenus *Passiflora* inhabit high elevations in the Andes Mountains, where cold acclimation is essential for survival. Another gene with strong positive selection in this subgenus is the *rbcL,* which encodes the large subunit of the ribulose-1,5-bisphosphate carboxylase/oxygenase (RuBisCO), a key enzyme in the Calvin cycle of photosynthesis [4]. Regarding positive selection of *rbcL*, it was demonstrated that stressful environments of the Mediterranean coast facilitated the diversification of Rubisco within the genus *Limonium*, resulting in different photosynthetic CO_2_ assimilation rates and plant growth responses to severe water stress [25]. Additionally, the adaptive evolution of the Rubisco L-subunit, particularly in response to climatic changes during the Oligocene and Miocene, was crucial for the diversification and environmental adaptability of species in the Brassicaceae [52].

Our study highlights that although purifying selection predominates in most chloroplast genes of *Passiflora*, some genes harbor sites under positive selection. Such adaptive evolution may contribute to the variability observed in this genus, including habitat diversity and morphological differences. Future research could further elucidate the specific environmental or functional drivers behind the positive selection of these cp genes, enhancing our understanding of plant adaptation and evolution.

### Nuclear phylogeny based on 35S rDNA sequences

We also generated a phylogeny based on the complete 18S and 26S rDNA sequences, which resulted in some well-supported clades, such as the monophyly of subgenera *Astrophea*. However, in general, this nuclear phylogeny showed lower bootstrap support values compared to the results from plastids. These differences can be attributed to the fact that the nuclear phylogeny was based only on two highly conserved markers, while the plastid trees were derived from a set of genes with varying levels of polymorphism. This expanded dataset in the plastids analysis likely enhanced the robustness in the phylogenetic resolution.

In plant phylogenetic studies, the ITS region of the 35S rDNA gene is a potential nuclear marker due to its high level of variation [3, 20, 36, 38]. Previously, the ITS region was used to reconstruct the first molecular *Passiflora* phylogenies [48, 61]. However, the very high levels of ITS variation could lead to the loss of phylogenetic signal by saturation, mainly in higher differentiated levels, as reported by Muschner et al. [61]. In this scenario, we decided to use the complete 18S and 26S rDNA genes, more conserved, which resulted in strong support for some nodes. Maia et al. [54] also suggested the potential of these gene sequences to infer phylogenies when analyzing a large sample of angiosperm species, but the low phylogenetic signal of this region resulted in low support for some clades. Aygoren Uluer et al. [5] used the 26S rDNA gene to infer interfamilial relationships in Fabales and noted a lack of support across the majority of nodes in the tree, especially for Leguminosae, as well as concerns regarding possible paralogy problems. In this way, the resulting trees of the 35S ribosomal cistron must be interpreted with caution, since this region occurs in multiple copies in the genome impacting the assembly of this region and the detection of paralogy. This is because it is challenging to determine the accurate homology for a single copy from 35S rDNA [23]. To address this issue, the use of nuclear low copy genes in plant phylogeny [31, 75, 98] would be an alternative to propose a nuclear phylogenetic hypothesis for *Passiflora*. For example, using target capture approaches and the Angiosperm 353 probe set, which targets single nuclear genes and have been shown to provide high-resolution phylogenetic trees [24, 57, 64].

## Conclusions

Our study reveals significant structural variations in the cp genomes of *Passiflora* species, elucidating their complex evolutionary relationships. These variations, including inversions, IR expansions and contractions, and differences in gene content, could be used as valuable markers to study the evolution of this genus. Gene sequence comparisons indicate that while some of the cp genes are under purifying selection, some genes, such as *clpP*, show evidence of positive selection, suggesting adaptive evolution. Additionally, the branch-site model test for positive selection revealed several genes under adaptive evolution specifically in subgenus *Passiflora*, including *atpA*, *atpB*, *ccsA*, *petL*, and *rbcL*. These findings highlight the potential adaptive roles which these genes played in the rapid radiation and ecological success of this subgenus. The phylogenetic analysis based on cp genomes provided a highly resolved tree, confirming the monophyly of the subgenera *Astrophea*, *Passiflora*, and *Decaloba*. In contrast, the nuclear 35S rDNA phylogeny did not provide much resolution for the tree, showing lower bootstrap support for many nodes within these subgenera. Notably, the polyphyletic position of subgenus *Deidamioides*, indicated by our findings, suggests the need for further studies and possibly a taxonomic revision to accurately reflect its evolutionary history.

## Supplementary Information

Supplementary Material 1.

Supplementary Material 2.

Supplementary Material 3.

## Data Availability

The data that support the findings of this study have been deposited in the NCBI database under the BioProject ID PRJNA1143559 and accession numbers SRR30189521—SRR30189530.

## Abbreviations

BS: Bootstrap
BP: Base pair
CP: Chloroplast
KB: Kilobase
PP: Posterior probability

## References

[1] Abeel T, Van Parys T, Saeys Y, Galagan J, Van De Peer Y. GenomeView: A next-generation genome browser. Nucleic Acids Res. 2012;40(2):e12. 10.1093/nar/gkr995. 22102585 10.1093/nar/gkr995 PMC3258165

[2] Abrahamczyk S, Souto-Vilarós D, Renner SS. Escape from extreme specialization: Passionflowers, bats and the sword-billed hummingbird. Proc R Soc B Biol Sci. 2014;281(1795):20140888. 10.1098/rspb.2014.0888. 10.1098/rspb.2014.0888 PMC4213610 25274372

[3] Álvarez I, Wendel JF. Ribosomal ITS sequences and plant phylogenetic inference. Mol Phylogenet Evol. 2003;29(3):417–34. 10.1016/S1055-7903(03)00208-2. 14615184 10.1016/s1055-7903(03)00208-2

[4] Andersson I, Backlund A. Structure and function of rubisco. Plant Physiol Biochem. 2008;46:275–91. 10.1016/j.plaphy.2008.01.001. 18294858 10.1016/j.plaphy.2008.01.001

[5] Aygoren Uluer D, Hawkins JA, Forest F. Interfamilial relationships in order Fabales: new insights from the nuclear regions sqd1 and 26S rDNA. Plant Syst Evol. 2020;306(4):66. 10.1007/s00606-020-01691-7.

[6] Beier S, Thiel T, Münch T, Scholz U, Mascher M. MISA-web: a web server for microsatellite prediction. Bioinformatics. 2017;33:2583–5. 10.1093/bioinformatics/btx198. 28398459 10.1093/bioinformatics/btx198 PMC5870701

[7] Buzato S, Franco ALM. *Tetrastylis ovalis*: A second case of bat-pollinated passionflower (Passifloraceae). Plant Syst Evol. 1992;181:261–7. 10.1007/BF00937450.

[8] Cao J, et al. Extreme Reconfiguration of Plastid Genomes in Papaveraceae: Rearrangements, Gene Loss, Pseudogenization, IR Expansion, and Repeats. Int J Mol Sci. 2024;25:2278. 10.3390/ijms25042278. 38396955 10.3390/ijms25042278 PMC10888665

[9] Cauz-Santos LA, da Costa ZP, Callot C, Cauet S, Zucchi MI, Bergès H, van den Berg C, Vieira MLC. A repertory of rearrangements and the loss of an inverted repeat region in *Passiflora* chloroplast genomes. Genome Biol Evol. 2020;12:1841–57. 10.1093/gbe/evaa155. 32722748 10.1093/gbe/evaa155 PMC7586853

[10] Cauz-Santos LA, Munhoz CF, Rodde N, Cauet S, Santos AA, Penha HA, Dornelas MC, Varani AM, Oliveira GCX, Bergès H, Vieira MLC. The chloroplast genome of *Passiflora edulis* (Passifloraceae) assembled from long sequence reads: structural organization and phylogenomic studies in Malpighiales. Front Plant Sci. 2017;8:1–17. 10.3389/fpls.2017.00334. 28344587 10.3389/fpls.2017.00334 PMC5345083

[11] Chumley TW, Palmer JD, Mower JP, Fourcade MH, Calie PJ, Boore JL, et al. The complete chloroplast genome sequence of Pelargonium × hortorum: organization and evolution of the largest and most highly rearranged chloroplast genome of land plants. Mol Biol Evol. 2006;23:2175–90. 10.1093/molbev/msl089. 10.1093/molbev/msl089 16916942

[12] Claude S-J, Park S, Park SJ. Gene loss, genome rearrangement, and accelerated substitution rates in plastid genome of *Hypericum ascyron* (Hypericaceae). BMC Plant Biol. 2022;22:135. 10.1186/s12870-022-03515-x. 35321651 10.1186/s12870-022-03515-x PMC8941745

[13] Cosner ME, Raubeson LA, Jansen RK. Chloroplast DNA rearrangements in Campanulaceae: Phylogenetic utility of highly rearranged genomes. BMC Evol Biol. 2004;4:27. 10.1186/1471-2148-4-27. 15324459 10.1186/1471-2148-4-27 PMC516026

[14] Daniell H, Lin CS, Yu M, Chang WJ. Chloroplast genomes: diversity, evolution, and applications in genetic engineering. Genome Biol. 2016;17:134. 10.1186/s13059-016-1004-2. 10.1186/s13059-016-1004-2 PMC4918201 27339192

[15] Darling AC, Mau B, Blattner FR, Perna NT. Mauve: multiple alignment of conserved genomic sequence with rearrangements. Genome Res. 2004;14:1394–403. 10.1101/gr.2289704. 10.1101/gr.2289704 PMC442156 15231754

[16] Dierckxsens N, Mardulyn P, Smits G. NOVOPlasty: De novo assembly of organelle genomes from whole genome data. Nucleic Acids Res. 2017;45(4):e18. 10.1093/nar/gkw955. 28204566 10.1093/nar/gkw955 PMC5389512

[17] Downie SR, Palmer JD. Use of chloroplast DNA rearrangements in reconstructing plant phylogeny. In: *Molecular Systematics of Plants*. Edited by Soltis PS, Soltis DE, Doyle JJ. Boston: Springer; 1992. 10.1007/978-1-4615-3276-7_2.

[18] Edgar RC. MUSCLE: Multiple sequence alignment with high accuracy and high throughput. Nucleic Acids Res. 2004;32:1792–7. 10.1093/nar/gkh340. 15034147 10.1093/nar/gkh340 PMC390337

[19] Erixon P, Oxelman B. Whole-gene positive selection, elevated synonymous substitution rates, duplication, and indel evolution of the chloroplast *clpP1* gene. PLoS ONE. 2008;3(1):e1386. 10.1371/journal.pone.0001386. 18167545 10.1371/journal.pone.0001386 PMC2148103

[20] Feng S, Jiang M, Shi Y, Jiao K, Shen C, Lu J, Ying Q, Wang H. Application of the ribosomal DNA ITS2 region of *Physalis* (Solanaceae): DNA barcoding and phylogenetic study. Front Plant Sci. 2016;7:1047. 10.3389/fpls.2016.01047. 27486467 10.3389/fpls.2016.01047 PMC4949264

[21] Feuillet C, MacDougal JM. A new infrageneric classification of *Passiflora* L. (Passifloraceae). Passiflora. 2003;13:34–5.

[22] Fleming TH, Geiselman C, Kress WJ. The evolution of bat pollination: A phylogenetic perspective. Ann Bot. 2009;104(6):1017–43. 10.1093/aob/mcp197. 19789175 10.1093/aob/mcp197 PMC2766192

[23] Fonseca LHM, Lohmann LG. Exploring the potential of nuclear and mitochondrial sequencing data generated through genome-skimming for plant phylogenetics: A case study from a clade of neotropical lianas. J Syst Evol. 2020;58:18–32. 10.1111/jse.12533.

[24] Fonseca LHM, Carlsen MM, Fine PVA, Lohmann LG. A nuclear target sequence capture probe set for phylogeny reconstruction of the charismatic plant family Bignoniaceae. Front Genet. 2023;13:1085692. 10.3389/fgene.2022.1085692. 36699458 10.3389/fgene.2022.1085692 PMC9869424

[25] Galmés J, Kapralov MV, Andralojc PJ, Conesa MÀ, Keys AJ, Parry MA, Flexas J. Expanding knowledge of the Rubisco kinetics variability in plant species: environmental and evolutionary trends. Plant Cell Environ. 2014;37:1989–2001. 10.1111/pce.12335. 24689692 10.1111/pce.12335

[26] Gao LZ, et al. Evolution of *Oryza* chloroplast genomes promoted adaptation to diverse ecological habitats. Commun Biol. 2019;2:278. 10.1038/s42003-019-0531-2. 31372517 10.1038/s42003-019-0531-2 PMC6659635

[27] Gao Y, Thiele W, Saleh O, Scossa F, Arabi F, Zhang H, Sampathkumar A, Kühn K, Fernie A, Bock R, Schöttler MA, Zoschke R. Chloroplast translational regulation uncovers nonessential photosynthesis genes as key players in plant cold acclimation. Plant Cell. 2022;34:2056–79. 10.1093/plcell/koac056. 35171295 10.1093/plcell/koac056 PMC9048916

[28] Greiner S, Lehwark P, Bock R. OrganellarGenomeDRAW (OGDRAW) version 1.3.1: expanded toolkit for the graphical visualization of organellar genomes. Nucleic Acids Res. 2019;47(W1):W59–64. 10.1093/nar/gkz238. 30949694 10.1093/nar/gkz238 PMC6602502

[29] Haberle RC, et al. Extensive rearrangements in the chloroplast genome of *Trachelium* caeruleum are associated with repeats and tRNA genes. J Mol Evol. 2008;66:350–61. 10.1007/s00239-008-9086-4. 18330485 10.1007/s00239-008-9086-4

[30] Henriquez CL, et al. Evolutionary dynamics of chloroplast genomes in subfamily Aroideae (Araceae). Genomics. 2020;112:2349–60. 10.1016/j.ygeno.2020.01.006. 31945463 10.1016/j.ygeno.2020.01.006

[31] Huang CH, Sun R, Hu Y, Zeng L, Zhang N, Cai L, Zhang Q, Koch MA, Al-Shehbaz I, Edger PP, Pires JC, Tan DY, Zhong Y, Ma H. Resolution of brassicaceae phylogeny using nuclear genes uncovers nested radiations and supports convergent morphological evolution. Mol Biol Evol. 2016;33(2):394–412. 10.1093/molbev/msv226. 26516094 10.1093/molbev/msv226 PMC4866547

[32] Ivanova Z, Sablok G, Daskalova E, Zahmanova G, Apostolova E, Yahubyan G, et al. Chloroplast genome analysis of resurrection tertiary relict Haberlea rhodopensis highlights genes important for desiccation stress response. Front Plant Sci. 2017;8:204. 10.3389/fpls.2017.00204. 10.3389/fpls.2017.00204 PMC5316520 28265281

[33] Jansen RK, Cai Z, Raubeson LA, Daniell H, Depamphilis CW, Leebens-Mack J, Müller KF, Guisinger-Bellian M, Haberle RC, Hansen AK, Chumley TW, Lee SB, Peery R, McNeal JR, Kuehl JV, Boore JL. Analysis of 81 genes from 64 plastid genomes resolves relationships in angiosperms and identifies genome-scale evolutionary patterns. Proc Natl Acad Sci USA. 2007;104(49):19369–74. 10.1073/pnas.0709121104. 18048330 10.1073/pnas.0709121104 PMC2148296

[34] Jansen RK, Palmer JD. A chloroplast DNA inversion marks an ancient evolutionary split in the sunflower family (Asteraceae). Proc Natl Acad Sci USA. 1987;84:5818–22. 10.1073/pnas.84.16.5818. 16593871 10.1073/pnas.84.16.5818 PMC298954

[35] Jiang D, Cai X, Gong M, et al. Complete chloroplast genomes provide insights into evolution and phylogeny of *Zingiber* (Zingiberaceae). BMC Genomics. 2023;24(1):30. 10.1186/s12864-023-09115-9. 36653780 10.1186/s12864-023-09115-9 PMC9848714

[36] Jobst J, King K, Hemleben V. Molecular evolution of the internal transcribed spacers (ITS1 and ITS2) and phylogenetic relationships among species of the family Cucurbitaceae. Mol Phylogenet Evol. 1998. 10.1006/mpev.1997.0465. 10.1006/mpev.1997.0465 9562980

[37] Kalyaanamoorthy S, Minh BQ, Wong TKF, Von Haeseler A, Jermiin LS. ModelFinder: Fast model selection for accurate phylogenetic estimates. Nat Methods. 2017. 10.1038/nmeth.4285. 10.1038/nmeth.4285 PMC5453245 28481363

[38] Käss E, Wink M. Molecular phylogeny and phylogeography of Lupinus (Leguminosae) inferred from nucleotide sequences of the rbcLgene and ITS 1 + 2 regions of rDNA. Plant Syst Evol. 1997;208:139–67. 10.1007/BF00985439.

[39] Katoh K, Standley DM. MAFFT multiple sequence alignment software version 7: Improvements in performance and usability. Mol Biol Evol. 2013;30:772–80. 10.1093/molbev/mst010. 23329690 10.1093/molbev/mst010 PMC3603318

[40] Killip EP. The American species of Passifloraceae. Chicago: Chicago Field Museum; 1938.

[41] Kirk PR, Leech RM. Amino acid biosynthesis by isolated chloroplasts during photosynthesis. Plant Physiol. 1972;50:228–34. 10.1104/pp.50.2.228. 16658147 10.1104/pp.50.2.228 PMC366115

[42] Kleffmann T, et al. The *Arabidopsis thaliana* chloroplast proteome reveals pathway abundance and novel protein functions. Curr Biol. 2004;14:354–62. 10.1016/j.cub.2004.02.039. 15028209 10.1016/j.cub.2004.02.039

[43] Knox EB. The dynamic history of plastid genomes in the Campanulaceae sensu lato is unique among angiosperms. Proc Natl Acad Sci USA. 2014;111:11097–102. 10.1073/pnas.1403363111. 25024223 10.1073/pnas.1403363111 PMC4121848

[44] Koren S, Walenz BP, Berlin K, Miller JR, Bergman NH, Phillippy AM. Canu: Scalable and accurate long-read assembly via adaptive κ-mer weighting and repeat separation. Genome Res. 2017. 10.1101/gr.215087.116. 10.1101/gr.215087.116 PMC5411767 28298431

[45] Krämer C, Boehm CR, Liu J, et al. Removal of the large inverted repeat from the plastid genome reveals gene dosage effects and leads to increased genome copy number. Nat Plants. 2024;10:923–35. 10.1038/s41477-024-01709-9. 38802561 10.1038/s41477-024-01709-9 PMC11208156

[46] Krosnick SE, Ford AJ, Freudenstein JV. Taxonomic revision of Passiflora subgenus Tetrapathea including the monotypic genera *Hollrungia* and *Tetrapathea* (Passifloraceae), and a new species of *Passiflora*. Syst Bot. 2009;34:375–85. 10.1600/036364409788606343.

[47] Krosnick SE, Freudenstein JV. Monophyly and floral character homology of Old world *Passiflora* (subgenus *Decaloba*: supersection *Disemma*). Syst Bot. 2005;30:139–52. 10.1600/0363644053661959.

[48] Krosnick SE, Porter-Utley KE, MacDougal JM, Jørgensen PM, McDade LA. New insights into the evolution of *Passiflora* subgenus *Decaloba* (Passifloraceae): Phylogenetic relationships and morphological synapomorphies. Syst Bot. 2013;38:692–713. 10.1600/036364413x670359.

[49] Kuroda H, Maliga P. The plastid *clpP1* protease gene is essential for plant development. Nature. 2003;425:86–9. 10.1038/nature01909. 12955146 10.1038/nature01909

[50] Kurtz S, Choudhuri JV, Ohlebusch E, Schleiermacher C, Stoye J, Giegerich R. REPuter: the manifold applications of repeat analysis on a genomic scale. Nucleic Acids Res. 2001;29:4633–42. 10.1093/nar/29.22.4633. 11713313 10.1093/nar/29.22.4633 PMC92531

[51] Lavin M, Doyle JJ, Palmer JD. Evolutionary significance of the loss of the chloroplast-DNA inverted repeat in the Leguminosae subfamily Papilionoideae. Evolution. 1990;44(2):390–402. 10.2307/2409416. 28564377 10.1111/j.1558-5646.1990.tb05207.x

[52] Liu L, Zhao B, Zhang Y, Wang J. Adaptive evolution of the *rbcL* gene in Brassicaceae. Biochem Syst Ecol. 2012;44:13–9. 10.1016/j.bse.2012.04.007.

[53] Ma PF, Zhang YX, Zeng CX, Guo ZH, Li DZ. Chloroplast phylogenomic analyses resolve deep-level relationships of an intractable bamboo tribe Arundinarieae (*Poaceae*). Syst Biol. 2014;63:933–50. 10.1093/sysbio/syu054. 25092479 10.1093/sysbio/syu054

[54] Maia VH, Gitzendanner MA, Soltis PS, Wong GKS, Soltis DE. Angiosperm phylogeny based on 18S/26S rDNA sequence data: Constructing a large data set using next-generation sequence data. Int J Plant Sci. 2014;175:613–50. 10.1086/676675.

[55] Maréchal A, Brisson N. Recombination and the maintenance of plant organelle genome stability. New Phytol. 2010;186:299–317. 10.1111/j.1469-8137.2010.03195.x. 20180912 10.1111/j.1469-8137.2010.03195.x

[56] Marinho LC, Cai L, Duan X, Ruhfel BR, Fiaschi P, Amorim AM, van den Berg C, Davis CC. Plastomes resolve generic limits within tribe *Clusieae* (Clusiaceae) and reveal the new genus Arawakia. Mol Phylogenet Evol. 2019;134:142–51. 10.1016/j.ympev.2019.02.005. 30743062 10.1016/j.ympev.2019.02.005

[57] McDonnell AJ, Baker WJ, Dodsworth S, Forest F, Graham SW, Johnson MG, Pokorny L, Tate J, Wicke S, Wickett NJ. Exploring Angiosperms353: Developing and applying a universal toolkit for flowering plant phylogenomics. Appl Plant Sci. 2021;9:11443. 10.1002/aps3.11443. 10.1002/aps3.11443 PMC8312743 34336400

[58] McMahon MM, Sanderson MJ. Phylogenetic supermatrix analysis of GenBank sequences from 2228 papilionoid legumes. Syst Biol. 2006;55(5):818–36. 10.1080/10635150600999150. 17060202 10.1080/10635150600999150

[59] Moore MJ, Bell CD, Soltis PS, Soltis DE. Using plastid genome-scale data to resolve enigmatic relationships among basal angiosperms. Proc Natl Acad Sci USA. 2007;104(49):19363–8. 10.1073/pnas.0708072104. 18048334 10.1073/pnas.0708072104 PMC2148295

[60] Moore MJ, Soltis PS, Bell CD, Burleigh JG, Soltis DE. Phylogenetic analysis of 83 plastid genes further resolves the early diversification of eudicots. Proc Natl Acad Sci USA. 2010;107(10):4623–8. 10.1073/pnas.0907801107. 20176954 10.1073/pnas.0907801107 PMC2842043

[61] Muschner VC, Lorenz AP, Cervi AC, Bonatto SL, Souza-Chies TT, Salzano FM, Freitas LB. A first molecular phylogenetic analysis of *Passiflora* (Passifloraceae). Am J Bot. 2003;90:1229–38. 10.3732/ajb.90.8.1229. 21659223 10.3732/ajb.90.8.1229

[62] Muschner VC, Zamberlan PM, Bonatto SL, Freitas LB. Phylogeny, biogeography and divergence times in *Passiflora* (Passifloraceae). Genet Mol Biol. 2012;35:1036–43. 10.1590/S1415-47572012000600019. 23412994 10.1590/s1415-47572012000600019 PMC3571420

[63] Ogihara Y, Terachi T, Sasakuma T. Intramolecular recombination of chloroplast genome mediated by short direct-repeat sequences in wheat species. Proc Natl Acad Sci USA. 1988;85:8573–7. 10.1073/pnas.85.22.8573. 3186748 10.1073/pnas.85.22.8573 PMC282501

[64] Ogutcen E, Christe C, Nishii K, Salamin N, Möller M, Perret M. Phylogenomics of Gesneriaceae using targeted capture of nuclear genes. Mol Phylogenet Evol. 2021;157:107068. 10.1016/j.ympev.2021.107068. 33422648 10.1016/j.ympev.2021.107068

[65] Oldenburg DJ, Bendich AJ. The linear plastid chromosomes of maize: terminal sequences, structures, and implications for DNA replication. Curr Genet. 2016;62:431–42. 10.1007/s00294-015-0548-0. 26650613 10.1007/s00294-015-0548-0

[66] Palmer JD, Nugent JM, Herbon LA. Unusual structure of geranium chloroplast DNA: A triple-sized inverted repeat, extensive gene duplications, multiple inversions, and two repeat families. Proc Natl Acad Sci USA. 1987;84:769–73. 10.1073/pnas.84.3.769. 16593810 10.1073/pnas.84.3.769 PMC304297

[67] Palmer JD, Thompson WF. Chloroplast DNA rearrangements are more frequent when a large inverted repeat sequence is lost. Cell. 1982;29:537–50. 10.1016/0092-8674(82)90170-2. 6288261 10.1016/0092-8674(82)90170-2

[68] Parks M, Cronn R, Liston A. Increasing phylogenetic resolution at low taxonomic levels using massively parallel sequencing of chloroplast genomes. BMC Biol. 2009;7:84. 10.1186/1741-7007-7-84. 19954512 10.1186/1741-7007-7-84 PMC2793254

[69] Rabah SO, Shrestha B, Hajrah NH, Sabir MJ, Alharby HF, Sabir MJ, Alhebshi AM, Sabir JSM, Gilbert LE, Ruhlman TA, Jansen RK. *Passiflora* plastome sequencing reveals widespread genomic rearrangements. J Syst Evol. (2019). 10.1111/jse.12425

[70] Rambaut A. FigTree v1.4.3. 2016. [http://tree.bio.ed.ac.uk/software/figtree/]. Accessed 10 May 2024.

[71] Raubeson LA, Jansen RK. Chloroplast genomes of plants. In: Plant Diversity and Evolution: Genotypic and Phenotypic Variation in Higher Plants. Edited by Henry R. London: CABI Publishing. 2005. p. 45–68. 10.1079/9780851999043.0045.

[72] Ruhlman TA, Zhang J, Blazier JC, Sabir JSM, Jansen RK. Recombination-dependent replication and gene conversion homogenize repeat sequences and diversify plastid genome structure. Am J Bot. 2017;104:559–72. 10.3732/ajb.1600453. 28400415 10.3732/ajb.1600453

[73] Ronquist F, Teslenko M, Van Der Mark P, Ayres DL, Darling A, Höhna S, Larget B, Liu L, Suchard MA, Huelsenbeck JP. MrBayes 3.2: Efficient Bayesian phylogenetic inference and model choice across a large model space. Syst Biol. 2012;61:539–42. 10.1093/sysbio/sys029. 10.1093/sysbio/sys029 PMC3329765 22357727

[74] Sader MA, Amorim BS, Costa L, Souza G, Pedrosa-Harand A. The role of chromosome changes in the diversification of *Passiflora* L. (Passifloraceae). Syst Biodivers. 2019;17:7–21. 10.1080/14772000.2018.1546777.

[75] Sang T. Utility of low-copy nuclear gene sequences in plant phylogenetics. Crit Rev Biochem Mol Biol. 2002;37:121–47. 10.1080/10409230290771474. 10.1080/10409230290771474 12139440

[76] Schneider AC, et al. Punctuated plastome reduction and host–parasite horizontal gene transfer in the holoparasitic plant genus Aphyllon. Proc R Soc B Biol Sci. 2018;285:20181535. 10.1098/rspb.2018.1535. 10.1098/rspb.2018.1535 PMC6170807 30232155

[77] Scobeyeva VA, Artyushin IV, Krinitsina AA, Nikitin PA, Antipin MI, Kuptsov SV, et al. Gene loss, pseudogenization in plastomes of genus Allium (Amaryllidaceae), and putative selection for adaptation to environmental conditions. Front Genet. 2021;12:674783. 10.3389/fgene.2021.674783. 10.3389/fgene.2021.674783 PMC8296844 34306019

[78] Shrestha B, Weng ML, Theriot EC, Gilbert LE, Ruhlman TA, Krosnick SE, Jansen RK. Highly accelerated rates of genomic rearrangements and nucleotide substitutions in plastid genomes of *Passiflora* subgenus *Decaloba*. Mol Phylogenet Evol. 2019;138:53–64. 10.1016/j.ympev.2019.05.030. 31129347 10.1016/j.ympev.2019.05.030

[79] Simmonds SE, et al. Phylogenetics and comparative plastome genomics of two of the largest genera of angiosperms, *Piper* and *Peperomia* (Piperaceae). Mol Phylogenet Evol. 2021;163:107229. 10.1016/j.ympev.2021.107229. 34129936 10.1016/j.ympev.2021.107229

[80] Shikanai T, Shimizu K, Ueda K, Nishimura Y, Kuroiwa T, Hashimoto T. The chloroplast *clpP* gene, encoding a proteolytic subunit of ATP-Dependent protease, is indispensable for chloroplast development in Tobacco. Plant Cell Physiol. 2001;42:264–73. 10.1093/pcp/pce031. 11266577 10.1093/pcp/pce031

[81] Stamatakis A. RAxML version 8: a tool for phylogenetic analysis and post-analysis of large phylogenies. Bioinformatics. 2014;30:1312–3. 10.1093/bioinformatics/btu033. 24451623 10.1093/bioinformatics/btu033 PMC3998144

[82] Stern DS, Higgs DC, Yang J. Transcription and translation in chloroplasts. Trends Plant Sci. 1997;2:308–15. 10.1016/S1360-1385(97)89953-0.

[83] Stirbet A, Lazár D, Guo Y, Govindjee G. Photosynthesis: basics, history and modelling. Ann Bot. 2020;126:511–37. 10.1093/aob/mcz171. 31641747 10.1093/aob/mcz171 PMC7489092

[84] Sugiura M. The chloroplast genome. Plant Mol Biol. 1992;19:149–68. 10.1007/BF00015612. 1600166 10.1007/BF00015612

[85] Takamatsu T, Baslam M, Inomata T, Oikawa K, Itoh K, Ohnishi T, et al. Optimized method of extracting rice chloroplast DNA for high-quality plastome resequencing and de novo assembly. Front Plant Sci. 2018;9:266. 10.3389/fpls.2018.00266. 10.3389/fpls.2018.00266 PMC5835797 29541088

[86] Tillich M, Lehwark P, Pellizzer T, Ulbricht-Jones ES, Fischer A, Bock R, Greiner S. GeSeq - Versatile and accurate annotation of organelle genomes. Nucleic Acids Res. 2017;45(W1):W6–11. 10.1093/nar/gkx391. 28486635 10.1093/nar/gkx391 PMC5570176

[87] Ulmer T, MacDougal JM. *Passiflora*: passionflowers of the world. Portland: Timber Press; 2004.

[88] Wang J, et al. Plant organellar genomes: Much done, much more to do. Trends Plant Sci. 2024;29(7):754–69. 10.1016/j.tplants.2023.12.014. 38220520 10.1016/j.tplants.2023.12.014

[89] Weng ML, Blazier JC, Govindu M, Jansen RK. Reconstruction of the ancestral plastid genome in Geraniaceae reveals a correlation between genome rearrangements, repeats, and nucleotide substitution rates. Mol Biol Evol. 2014;31:645–59. 10.1093/molbev/mst257. 10.1093/molbev/mst257 24336877

[90] Williams AV, Boykin LM, Howell KA, Nevill PG, Small I. Correction: The Complete Sequence of the *Acacia ligulata* Chloroplast Genome Reveals a Highly Divergent clpP1 Gene. PLoS ONE. 2015;10(9):e0138367. 10.1371/journal.pone.0138367. 26367530 10.1371/journal.pone.0138367 PMC4569417

[91] Wojciechowski MF, Lavin M, Sanderson MJ. A phylogeny of legumes (Leguminosae) based on analysis of the plastid *matK* gene resolves many well-supported subclades within the family. Am J Bot. 2004;91(11):1846–62. 10.3732/ajb.91.11.1846. 21652332 10.3732/ajb.91.11.1846

[92] Wortley AH, Rudall PJ, Harris DJ, Scotland RW. How much data are needed to resolve a difficult phylogeny?: case study in Lamiales. Syst Biol. 2005;54(5):697–709. 10.1080/10635150500221028. 16195214 10.1080/10635150500221028

[93] Wu S, Chen J, Li Y, et al. Extensive genomic rearrangements mediated by repetitive sequences in plastomes of *Medicago* and its relatives. BMC Plant Biol. 2021;21:421. 10.1186/s12870-021-03202-3. 34521343 10.1186/s12870-021-03202-3 PMC8438982

[94] Xi Z, Ruhfel BR, Schaefer H, Amorim AM, Sugumaran M, Wurdack KJ, Endress PK, Matthews ML, Stevens PF, Mathews S, Davis CC. Phylogenomics and a posteriori data partitioning resolve the Cretaceous angiosperm radiation Malpighiales. Proc Natl Acad Sci USA. 2012;109:17519–24. 10.1073/pnas.1205818109. 23045684 10.1073/pnas.1205818109 PMC3491498

[95] Yang Z. Likelihood ratio tests for detecting positive selection and application to primate lysozyme evolution. Mol Biol Evol. 1998;15:568–73. 10.1093/oxfordjournals.molbev.a025957. 9580986 10.1093/oxfordjournals.molbev.a025957

[96] Yang Z, Nielsen R. Codon-substitution models for detecting molecular adaptation at individual sites along specific lineages. Mol Biol Evol. 2002;19:908–17. 10.1093/oxfordjournals.molbev.a004148. 12032247 10.1093/oxfordjournals.molbev.a004148

[97] Yockteng R, Nadot S. Phylogenetic relationships among *Passiflora* species based on the glutamine synthetase nuclear gene expressed in chloroplast (*ncpGS)*. Mol Phylogenet Evol. 2004;31(1):379–96. 10.1016/S1055-7903(03)00277-X. 15019632 10.1016/S1055-7903(03)00277-X

[98] Zhang N, Zeng L, Shan H, Ma H. Highly conserved low-copy nuclear genes as effective markers for phylogenetic analyses in angiosperms. New Phytol. 2012;195(4):923–37. 10.1111/j.1469-8137.2012.04212.x. 22783877 10.1111/j.1469-8137.2012.04212.x

[99] Zhang SD, Jin JJ, Chen SY, Chase MW, Soltis DE, Li HT, Yang JB, Li DZ, Yi TS. Diversification of Rosaceae since the Late Cretaceous based on plastid phylogenomics. New Phytol. 2017;214(3):1355–67. 10.1111/nph.14461. 28186635 10.1111/nph.14461

[100] Zhang YJ, Ma PF, Li DZ. High-throughput sequencing of six bamboo chloroplast genomes: Phylogenetic implications for temperate woody bamboos (*Poaceae*: Bambusoideae). PLoS ONE. 2011;6(5):e20596. 10.1371/journal.pone.0020596. 21655229 10.1371/journal.pone.0020596 PMC3105084

[101] Zhang Y, Zhang A, Li X, Lu C. The role of chloroplast gene expression in plant responses to environmental stress. Int J Mol Sci. 2020;21:6082. 10.3390/ijms21176082. 32846932 10.3390/ijms21176082 PMC7503970

[102] Zhao F, Chen YP, Salmaki Y, et al. An updated tribal classification of Lamiaceae based on plastome phylogenomics. BMC Biol. 2021;19:2. 10.1186/s12915-020-00931-z. 33419433 10.1186/s12915-020-00931-z PMC7796571

[103] Zheng B, Halperin T, Hruskova-Heidingsfeldova O, Adam Z, Clarke AK. Characterization of Chloroplast Clp proteins in Arabidopsis: Localization, tissue specificity and stress responses. Physiol Plant. 2002;114:92–101. 10.1034/j.1399-3054.2002.1140113.x. 11982939 10.1034/j.1399-3054.2002.1140113.x

[104] Zhou XM, Wang J, Li F, Li Q, Hu WP, Yang CP, et al. Inferring the evolutionary mechanism of the chloroplast genome size by comparing whole-chloroplast genome sequences in seed plants. Sci Rep. 2017;7:1555. 10.1038/s41598-017-01518-5. 10.1038/s41598-017-01518-5 PMC5431534 28484234

