# Supplementary material for: Chloroplast genomic insights into adaptive evolution and rapid radiation in the genus Passiflora (Passifloraceae)
Source: BMC Plant Biol. 2025 Feb 13;25:192. doi: 10.1186/s12870-025-06210-9 (PMC11823247; doi:10.1186/s12870-025-06210-9)

**Supplementary Figure 1**. Chloroplast genome maps of 11 *Passiflora* species obtained in this study.

Chloroplast genome map of *Passiflora rusbyi*, subgenus *Astrophea*. Genes are displayed as boxes inside or outside the main circle to indicate clockwise (inside) or counter-clockwise (outside) transcription. Gene boxes are color-coded based on their functional groups. The thickened segments of the main circle indicate the IR regions. The innermost circle represents the GC content across the genome. LSC refers to the large single-copy region, and SSC refers to the small single-copy region.

Chloroplast genome map of *Passiflora tetrandra*, subgenus *Tetraphatea*. Genes are displayed as boxes inside or outside the main circle to indicate clockwise (inside) or counter-clockwise (outside) transcription. Gene boxes are color-coded based on their functional groups. The thickened segments of the main circle indicate the IR regions. The innermost circle represents the GC content across the genome. LSC refers to the large single-copy region, and SSC refers to the small single-copy region.

Chloroplast genome map of *Passiflora adenopoda*, subgenus *Decaloba*. Genes are displayed as boxes inside or outside the main circle to indicate clockwise (inside) or counter-clockwise (outside) transcription. Gene boxes are color-coded based on their functional groups. The thickened segments of the main circle indicate the IR regions. The innermost circle represents the GC content across the genome. LSC refers to the large single-copy region, and SSC refers to the small single-copy region.

Chloroplast genome map of *Passiflora intricata*, subgenus *Decaloba*. Genes are displayed as boxes inside or outside the main circle to indicate clockwise (inside) or counter-clockwise (outside) transcription. Gene boxes are color-coded based on their functional groups. The thickened segments of the main circle indicate the IR regions. The innermost circle represents the GC content across the genome. LSC refers to the large single-copy region, and SSC refers to the small single-copy region.

Chloroplast genome map of *Passiflora xiikzdoz*, subgenus *Decaloba*. Genes are displayed as boxes inside or outside the main circle to indicate clockwise (inside) or counter-clockwise (outside) transcription. Gene boxes are color-coded based on their functional groups. The thickened segments of the main circle indicate the IR regions. The innermost circle represents the GC content across the genome. LSC refers to the large single-copy region, and SSC refers to the small single-copy region.

Chloroplast genome map of *Passiflora chaparensis*, subgenus *Passiflora*. Genes are displayed as boxes inside or outside the main circle to indicate clockwise (inside) or counter-clockwise (outside) transcription. Gene boxes are color-coded based on their functional groups. The thickened segments of the main circle indicate the IR regions. The innermost circle represents the GC content across the genome. LSC refers to the large single-copy region, and SSC refers to the small single-copy region.

Chloroplast genome map of *Passiflora garckei*, subgenus *Passiflora*. Genes are displayed as boxes inside or outside the main circle to indicate clockwise (inside) or counter-clockwise (outside) transcription. Gene boxes are color-coded based on their functional groups. The thickened segments of the main circle indicate the IR regions. The innermost circle represents the GC content across the genome. LSC refers to the large single-copy region, and SSC refers to the small single-copy region.

Chloroplast genome map of *Passiflora palenquensis*, subgenus *Passiflora*. Genes are displayed as boxes inside or outside the main circle to indicate clockwise (inside) or counter-clockwise (outside) transcription. Gene boxes are color-coded based on their functional groups. The thickened segments of the main circle indicate the IR regions. The innermost circle represents the GC content across the genome. LSC refers to the large single-copy region, and SSC refers to the small single-copy region.

Chloroplast genome map of *Passiflora phoenicea*, subgenus *Passiflora*. Genes are displayed as boxes inside or outside the main circle to indicate clockwise (inside) or counter-clockwise (outside) transcription. Gene boxes are color-coded based on their functional groups. The thickened segments of the main circle indicate the IR regions. The innermost circle represents the GC content across the genome. LSC refers to the large single-copy region, and SSC refers to the small single-copy region.

Chloroplast genome map of *Passiflora popenovii*, subgenus *Passiflora*. Genes are displayed as boxes inside or outside the main circle to indicate clockwise (inside) or counter-clockwise (outside) transcription. Gene boxes are color-coded based on their functional groups. The thickened segments of the main circle indicate the IR regions. The innermost circle represents the GC content across the genome. LSC refers to the large single-copy region, and SSC refers to the small single-copy region.

Chloroplast genome map of *Passiflora racemosa*, subgenus *Passiflora*. Genes are displayed as boxes inside or outside the main circle to indicate clockwise (inside) or counter-clockwise (outside) transcription. Gene boxes are color-coded based on their functional groups. The thickened segments of the main circle indicate the IR regions. The innermost circle represents the GC content across the genome. LSC refers to the large single-copy region, and SSC refers to the small single-copy region.

**Supplementary Figure 2**. Gene arrangements at the junctions of the quadripartite structure of cp genomes in different *Passiflora* subgenera. Each species is shown with the gene order at the junctions between the large single-copy (LSC), small single-copy (SSC), and inverted repeat (IR) regions. The gene boxes indicate the relative positions and orientations of genes at the junctions.


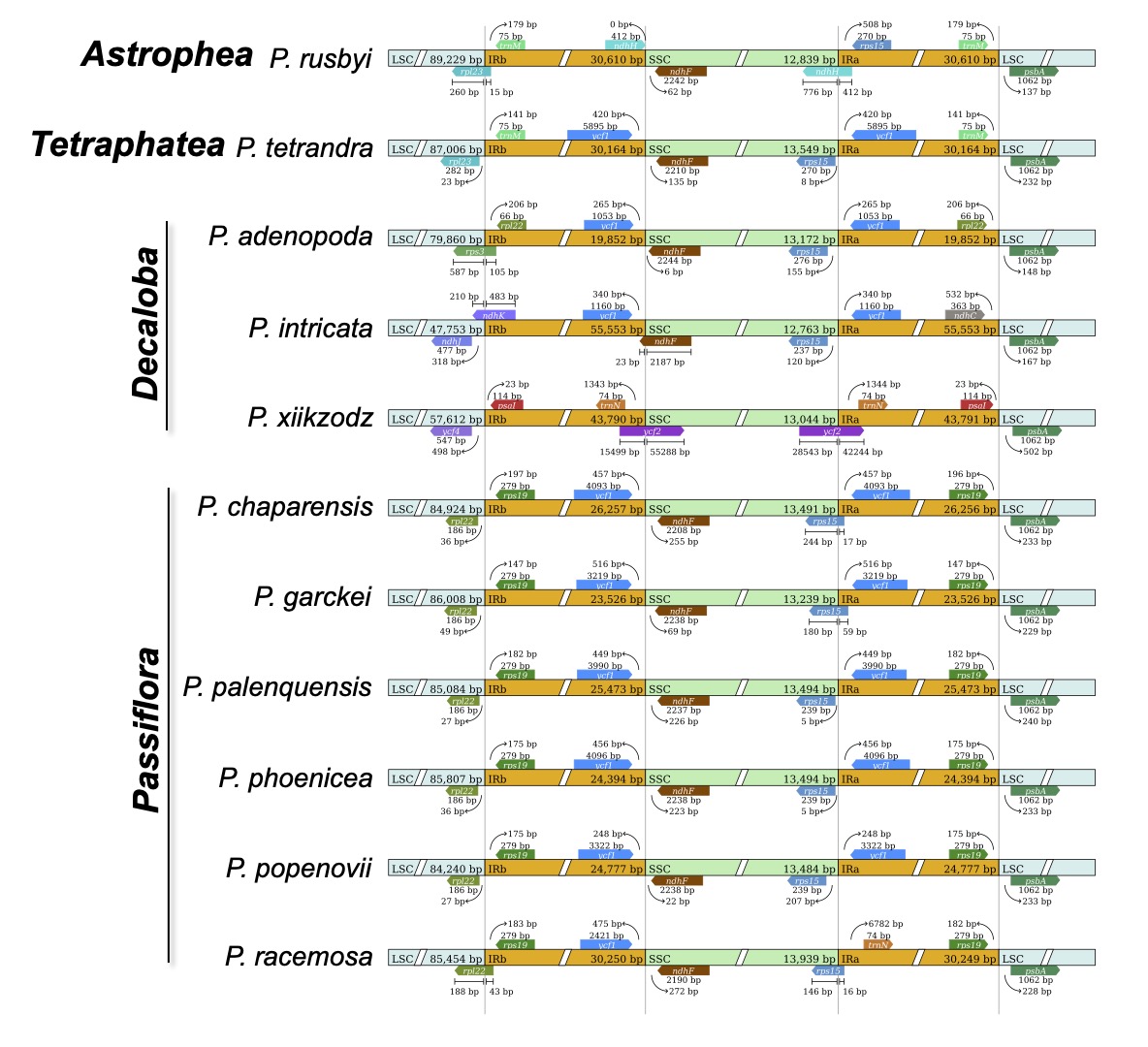


**Supplementary Figure 3**. Bayesian phylogenetic tree obtained from whole cp genome sequences of the subgenus *Passiflora*. The colors of the taxon’s indicate the supersections classification*.*


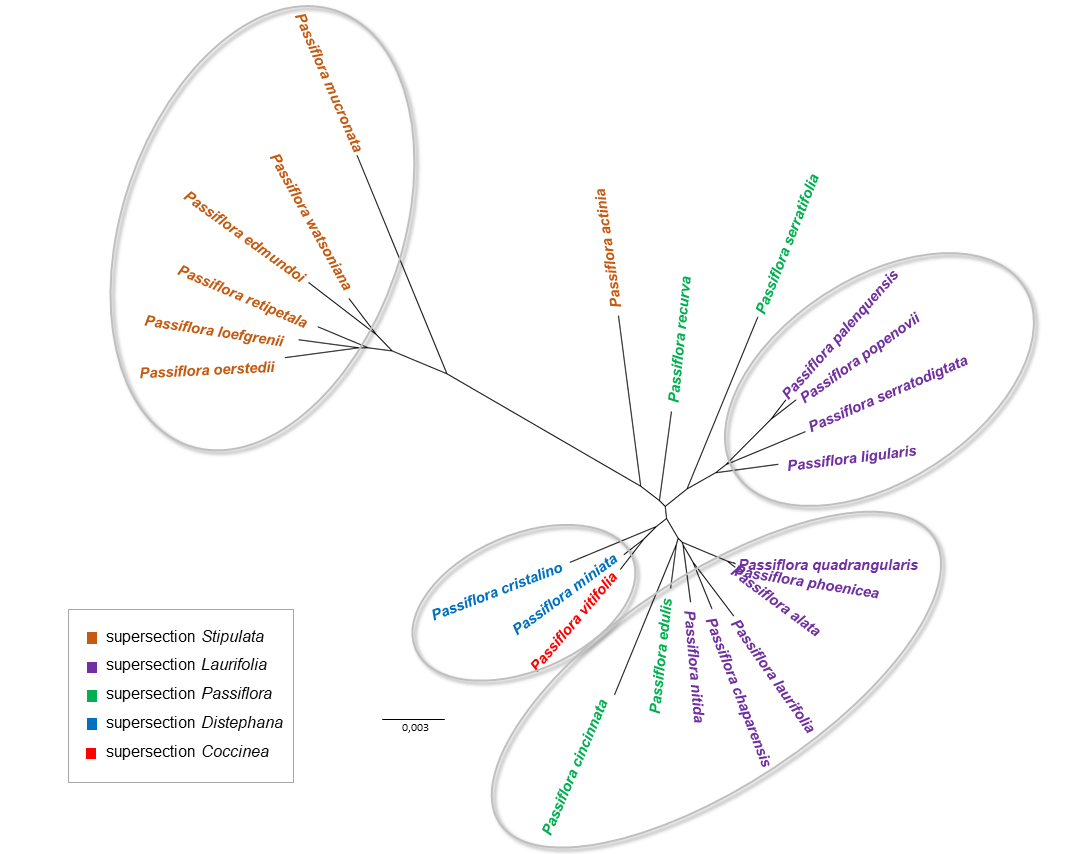

Supplement: Supplementary file 1 — Supplementary Material 1. [file 12870_2025_6210_MOESM1_ESM.docx]
