# Supplementary material for: Chloroplast genomic insights into adaptive evolution and rapid radiation in the genus Passiflora (Passifloraceae)
Source: BMC Plant Biol. 2025 Feb 13;25:192. doi: 10.1186/s12870-025-06210-9 (PMC11823247; doi:10.1186/s12870-025-06210-9)
Supplement: Supplementary file 2 — Supplementary Material 2. [file 12870_2025_6210_MOESM2_ESM.docx]

**Supplementary Table 1.** List of NCBI’s Genbank accession numbers for the chloroplast genomes of *Passiflora* species used in the phylogenomic analysis, as well the outgroups from other Passifloraceae species and *Populus trichocarpa*.

| **Subgenus** | **Species** | **Data** | **Plastid Genes** | **Whole Plastid** | **18S/26S** |
| --- | --- | --- | --- | --- | --- |
| ***Astrophea*** | *Passiflora cerradensis* | MT525871 | X |  | X |
|  | *Passiflora haematostigma* | MT525875 | X |  | X |
|  | *Passiflora pittieri* | NC_038125.1 | X |  |  |
|  | *Passiflora rhamnifolia* | MT525882 | X |  | X |
|  | *Passiflora rusbyi* | This study | X |  | X |
| ***Decaloba*** | *Passiflora adenopoda* | This study | X |  | X |
|  | *Passiflora affinis* | NC_043823.1 | X |  |  |
|  | *Passiflora auriculata* | NC_038119.1 | X |  |  |
|  | *Passiflora biflora* | NC_038120.1 | X |  |  |
|  | *Passiflora candollei* | MT525870 | X |  | X |
|  | *Passiflora capsularis* | MT525883 | X |  | X |
|  | *Passiflora costaricensis* | MT473979 | X |  | X |
|  | *Passiflora filipes* | NC_043822.1 | X |  |  |
|  | *Passiflora intricata* | This study | X |  | X |
|  | *Passiflora jatunsachensis* | NC_043813.1 | X |  |  |
|  | *Passiflora lutea* | NC_043815.1 | X |  |  |
|  | *Passiflora microstipula* | NC_043827.1 | X |  |  |
|  | *Passiflora misera* | NC_043821.1 | X |  |  |
|  | *Passiflora organensis* | JAEPBF000000000 | X |  | X |
|  | *Passiflora rufa* | NC_043817.1 | X |  |  |
|  | *Passiflora suberosa* | MT525868 | X |  | X |
|  | *Passiflora tenuiloba* | NC_043816.1 | X |  |  |
|  | *Passiflora vespertilio* | MT525880 | X |  | X |
|  | *Passiflora xiikzozdz* | This study | X |  | X |
| ***Deidamioides*** | *Passiflora arbelaezii* | NC_043819.1 | X |  |  |
|  | *Passiflora contracta* | MT533196 | X |  | X |
|  | *Passiflora deidamioides* | MT525873 | X |  | X |
|  | *Passiflora obovata* | NC_043824.1 | X |  |  |
| ***Tetraphatea*** | *Passiflora tetrandra* | This study | X |  | X |
| ***Passiflora*** | *Passiflora actinia* | NC_038118.1 | X | X |  |
|  | *Passiflora alata* | MT525869 | X | X | X |
|  | *Passiflora chaparensis* | This study | X | X | X |
|  | *Passiflora cincinnata* | NC_037690.1 | X | X |  |
|  | *Passiflora cristalina* | MT525872 | X | X | X |
|  | *Passiflora edmundoi* | MT525874 | X | X | X |
|  | *Passiflora edulis* | NC_034285.1 | X | X |  |
|  | *Passiflora foetida* | NC_043825.1 | X | X |  |
|  | *Passiflora garckei* | This study | X | X | X |
|  | *Passiflora laurifolia* | NC_038121.1 | X | X |  |
|  | *Passiflora ligularis* | NC_038122.1 | X | X |  |
|  | *Passiflora loefgrenii* | MT525876 | X | X | X |
|  | *Passiflora menispermifolia* | NC_043826.1 | X | X |  |
|  | *Passiflora miniata* | MT525877 | X | X | X |
|  | *Passiflora mucronata* | MT525878 | X | X | X |
|  | *Passiflora nitida* | NC_038123.1 | X | X |  |
|  | *Passiflora oerstedii* | NC_038124.1 | X | X |  |
|  | *Passiflora palenquensis* | This study | X | X | X |
|  | *Passiflora phoenicea* | This study | X | X | X |
|  | *Passiflora popenovii* | This study | X | X | X |
|  | *Passiflora quadrangularis* | NC_038126.1 | X | X |  |
|  | *Passiflora racemosa* | This study | X | X |  |
|  | *Passiflora recurva* | MT525879 | X | X | X |
|  | *Passiflora retipetala* | NC_038188.1 | X | X |  |
|  | *Passiflora serratifolia* | NC_038129.1 | X | X |  |
|  | *Passiflora serratodigitata* | NC_038127.1 | X | X |  |
|  | *Passiflora vitifolia* | NC_038128.1 | X | X |  |
|  | *Passiflora watsoniana* | MT525881 | X | X | X |
| **Passifloraceae** | *Adenia mannii* | NC_043791.1 | X |  |  |
|  | *Dilkea retusa* | MT525866 | X |  | X |
|  | *Mitostemma brevifilis* | MT525867 | X |  | X |
| **Outgroup** | *Populus trichocarpa* | NC_009143.1 | X |  |  |
